# Marker Recycling in *Candida albicans* through CRISPR-Cas9-Induced Marker Excision

**DOI:** 10.1128/mSphere.00050-17

**Published:** 2017-03-15

**Authors:** Manning Y. Huang, Aaron P. Mitchell

**Affiliations:** Department of Biological Sciences, Carnegie Mellon University, Pittsburgh, Pennsylvania, USA; Yonsei University

**Keywords:** CRISPR, biotechnology, genetics

## Abstract

We describe here a new approach to marker recycling, a controlled sequence of steps in which a genetic marker is selected and then lost. Marker recycling is important for genetic manipulation, because it allows a single selection marker to be used repeatedly. Our approach relies upon the ability of the CRISPR-Cas9 system to make a targeted double-strand break in DNA and the expectation that a double-strand break within a selection marker may promote recombination between directly repeated sequences that flank the marker. We call the approach CRISPR-Cas9-induced marker excision (CRIME). We tested the utility of this approach with the fungal pathogen *Candida albicans*, which is typically diploid. We used two selection markers, modified to include flanking direct repeats. In a proof-of-principle study, we created successive homozygous deletions in three genes through use of the two markers and had one of the markers available in the final strain for further selection and recycling. This strategy will accelerate the creation of multiple-mutant strains in *C. albicans*. CRISPR-Cas9 systems have been applied to many organisms, so the genetic design principles described here may be broadly applicable.

**IMPORTANCE** It is critical to be able to alter genes in order to elucidate their functions. These alterations often rely upon markers that allow selection for a rare cell in a population that has incorporated a piece of DNA. The number of alterations that can be accomplished is thus limited by the number of selection markers that are available. This limitation is circumvented by marker recycling strategies, in which a marker is eliminated after its initial use. Then, the marker can be used again. In this report, we describe a new marker recycling strategy that is enabled by recently developed CRISPR-Cas9 technology.

## Abstract (precis)

It is critical to be able to alter genes in order to elucidate their functions. These alterations often rely upon markers that allow selection for a rare cell in a population that has incorporated a piece of DNA. The number of alterations that can be accomplished is thus limited by the number of selection markers that are available. This limitation is circumvented by marker recycling strategies, in which a marker is eliminated after its initial use. Then, the marker can be used again. In this report, we describe a new marker recycling strategy that is enabled by recently developed CRISPR-Cas9 technology.

## INTRODUCTION

Engineered genetic manipulations almost always require selection markers, and for many organisms only a few markers are useful. The spectrum of selection markers may be limited by an organism’s intrinsic resistance to drugs, the complexity of medium formulations, phenotypic impact of a growth requirement, or other factors. Therefore, it is helpful to be able to use a single selection marker repeatedly. The repeated use of the same marker for genetic constructs that are integrated stably in the genome is achieved through an approach called marker recycling, in which a strategy to promote or detect loss of a marker can be applied after the initial selection for the marker.

Marker recycling has been achieved through two general approaches: positive/negative selection or recombinase-promoted excision. In the positive/negative selection approach, a marker cassette is used that permits growth under one condition and prevents growth under another condition. The cassette includes flanking directly repeated sequences that allow low-frequency homologous recombination events to excise the marker, leaving behind one copy of the repeated sequence. This approach was popularized with the development of the “Ura-blaster” for *Saccharomyces cerevisiae*, which was rapidly adapted for use in other fungi (1, 2). In the recombinase-promoted excision approach, the marker cassette includes both a selection marker and an inducible site-specific recombinase gene. Target sites for the recombinase lie at the ends of the cassette, so that induction of the recombinase causes high-frequency excision of the marker cassette. In essence, the activity of the recombinase increases the cassette excision rate sufficiently so that no selection against the marker cassette is necessary to detect loss events. This approach was popularized with the development of the “*SAT1* flipper” in *Candida albicans* (3) and is related to the Cre-lox system, which is used to create conditional knockouts in mice (4).

Here, we present a marker recycling approach that builds upon CRISPR-Cas9 systems. These systems use a programmable nuclease to make a targeted double-strand break in DNA (5). Targeting is accomplished by base-pairing between one genomic DNA strand and the single-guide RNA (sgRNA) that is complexed with the Cas9 nuclease (5, 6). An investigator can choose the site at which a double-strand break will be induced by designing an appropriate sgRNA. We applied our approach to the fungal pathogen *C. albicans* for proof of principle. *C. albicans* is extremely important clinically (7) and presents challenging genetics because it is naturally diploid and lacks a complete sexual cycle (8). In most cases, a recessive loss-of-function mutation must be homozygous in order to manifest a prominent phenotype, so gene function analysis in this organism has typically required at least two successive transformations. The creation of homozygous mutants was accelerated dramatically through the work of Vyas et al., who developed a CRISPR-Cas system for *C. albicans* (9). They showed that homozygous mutations in one or even several genes could be created in a single transformation. We previously modified their system to create complete gene deletion mutations, and we found that the genes specifying Cas9 and the sgRNA could be introduced into cells transiently and without direct selection (10). The marker recycling approach we describe here was tested specifically in *C. albicans* and is based upon the general properties of CRISPR-Cas9 systems and the native recombination and repair machinery of the cell. Because CRISPR-Cas9 systems have been deployed in a broad spectrum of organisms, we believe that our marker recycling strategy may be generally useful.

## RESULTS

### Rationale for CRISPR-Cas9-induced marker excision.

**FIG 1:**
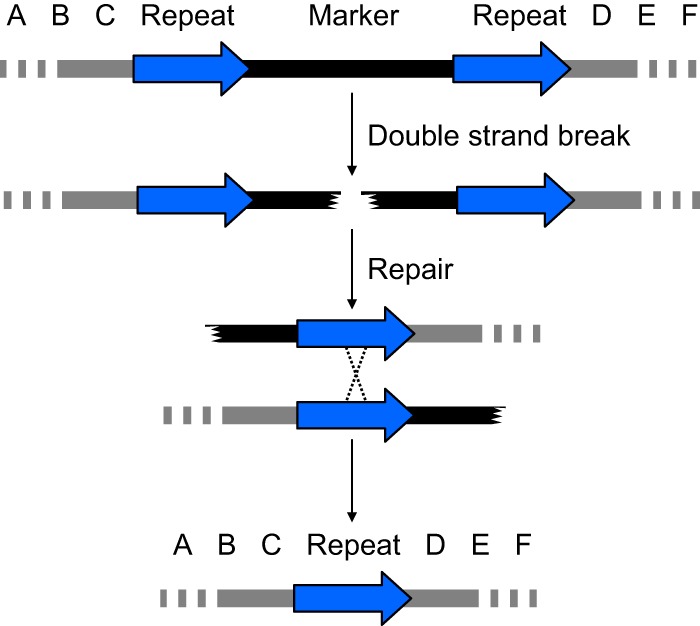
Break-induced marker excision concept. A selection marker is flanked by directly repeated sequences (blue arrows) in the genomic region designated “A B C D E F” (top line). A double-strand break within the marker (second line) results in a recombination event between the repeats (third line) that, when resolved, deletes the marker (fourth line). This type of excision reaction was shown to occur *in vivo* in *S. cerevisiae* by Sugawara and Haber (11). Mechanistically, the recombination event may occur through the single-strand annealing pathway, the microhomology-mediated end-joining pathway, or other homology-promoted repair events (21). A textbook-style crossover is depicted for simplicity of visualization.

A double-strand break in a genomic region flanked by directly repeated sequences should yield a deletion that fuses the flanking repeats (Fig. 1). This expectation is founded on the pioneering study by Sugawara and Haber of break-induced recombination (11). We reasoned that this recombination process should allow loss, through excision, of any selection marker. Moreover, if a cell was homozygous for the entire region depicted, then both alleles could undergo the same recombination process, provided that both alleles were subjected to a double-strand break.

**FIG 2:**
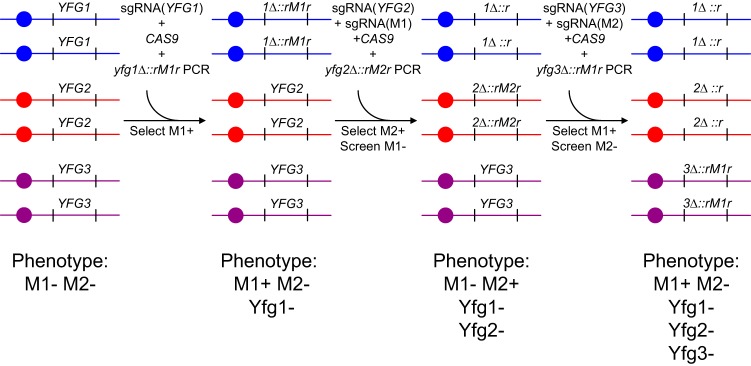
Strategy for marker recycling through CRISPR-Cas9-induced marker excision. Consider a situation in which an investigator seeks to make a *C. albicans* strain with homozygous deletion mutations in three genes—*YFG1*, *YFG2*, and *YFG3*—using only two selection markers, *M1* and *M2*. The marker cassettes, modified to include flanking direct repeats, are designated *rM1r* and *rM2r*. The three *YFG* genes are shown on separate blue, red, and violet chromosomes. The construction is carried out with only three transformations. For transformation 1, first a homozygous *yfg1*Δ::*rM1r* mutant is created through transformation of the strain with a *yfg1*Δ::*rM1r* PCR product, an sgRNA gene that targets *YFG1*, and a *CAS9* gene. The M1^+^ phenotype is selected. For transformation 2, after genotyping, a homozygous *yfg1*Δ::*rM1r* mutant is chosen and transformed to create a homozygous *yfg2*Δ::*rM2r* mutation. The transformation mix includes a *yfg2*Δ::*rM2r* PCR product, an sgRNA gene that targets *YFG2*, and a *CAS9* gene. In addition, in order to eliminate the *M1* marker by recombination between flanking repeats, an sgRNA gene that targets *M1* itself is also included. For this transformation, the M2^+^ phenotype is selected. Among M2^+^ transformants, some are M1^−^. The M1^−^ transformants are genotyped to identify homozygous *yfg2*Δ::*rM2r* mutants. In addition, PCR genotyping is used to verify that *yfg1*Δ::*r* is homozygous, marked only with a repeat sequence and not with the entire *M1* marker. For transformation 3, a strain homozygous for *yfg1*Δ::*r yfg2*Δ::*rM2r* is chosen, and the strain is transformed to create a homozygous *yfg3*Δ::*rM1r* mutation. The transformation mix includes a *yfg3*Δ::*rM1r* PCR product, an sgRNA gene that targets *YFG3*, and a *CAS9* gene. In addition, in order to eliminate the *M2* marker by recombination between flanking repeats, an sgRNA gene that targets *M2* itself is also included. For this transformation, the M1^+^ phenotype is selected once again, just as it was in the initial transformation. Among M1^+^ transformants, some are M2^−^. The M2^−^ transformants are genotyped to identify homozygous *yfg3*Δ::*rM1r* mutants. In addition, PCR genotyping is used to verify that *yfg2*Δ::*r* is homozygous, marked only with a repeat sequence and not with the entire *M2* marker.

Such marker excision events could be implemented with the use of CRISPR-Cas9 to create a marker recycling system. Consider that an investigator seeks to make a *C. albicans* strain with homozygous deletion mutations in three genes—*YFG1*, *YFG2*, and *YFG3*—and can use only two selection markers, *M1* and *M2*. The specific marker cassettes would include flanking direct repeats, and we would call the cassettes *rM1r* and *rM2r*. The construction of the homozygous triple mutant could be accomplished in three successive transformations (Fig. 2). In the first transformation, the *YFG1* gene is replaced with *yfg1Δ*::*rM1r* at both alleles. Biallelic replacement is accomplished by including in the transformation mix the genes that specify Cas9 and a *YFG1-*targeting sgRNA along with the *yfg1Δ*::*rM1r* repair template. The second transformation is carried out with the strain that resulted from the first transformation. In the second transformation, the *YFG2* gene is replaced with *yfg2Δ*::*rM2r* at both alleles; in addition, the *rM1r* marker is excised to leave behind *yfg1Δ*::*r* at both *yfg1Δ* alleles. These two biallelic events are accomplished by including in the transformation mix the genes that specify Cas9, a *YFG2-*targeting sgRNA, and an *M1*-targeting sgRNA, along with the *yfg2Δ*::*rM2r* repair template. Hence, the resulting strain lacks the *M1* marker, so that the marker can be used for selection again. This strain is used for the third transformation. In the third transformation, the *YFG3* gene is replaced with *yfg3Δ*::*rM1r* at both alleles; in addition, the *rM2r* marker is excised to leave behind *yfg2Δ*::*r* at both *yfg2Δ* alleles. These two biallelic events are accomplished analogously to those of the second transformation. Specifically, the transformation mix includes genes that specify Cas9, a *YFG3-*targeting sgRNA, and an *M2*-targeting sgRNA, along with the *yfg3Δ*::*rM1r* repair template. These three transformations yield a *yfg1 yfg2 yfg3* homozygous triple-deletion mutant that carries the *M1* marker but lacks the *M2* marker, so that the *M2* marker can be used for selection again. We refer to each marker excision step (the conversion of *rM1r* to *r*, or the conversion of *rM2r* to *r*) as CRISPR-Cas9-induced marker excision, which we abbreviate CRIME.

### Application of CRIME.

To see whether CRIME works in practice, we set out to create a homozygous *ume6Δ brg1Δ bcr1Δ* triple mutant. Each of the genes chosen for deletion is a positive regulator of filamentation and biofilm formation (12–14). We used the popular strain SN152, which is homozygous for the mutations *his1*Δ and *leu2*Δ (15). (It is also homozygous for *arg4*Δ, but we did not use the *ARG4* gene in our studies.) The *yfgΔ*::*rMr* repair templates comprised two overlapping PCR products (each with a single “r” repeat sequence, to create a split-marker template [16], as detailed in Materials and Methods). (Note that our split-marker transformations include two overlapping fragments of the repair template, and their final assembly requires cellular recombination machinery.) There were different flanking repeat sequences for *r1HIS1r1* and *r2LEU2r2*, in order to minimize the possibility of recombinational interaction between the cassettes. The *r1HIS1r1* marker included flanking repeats of 360 bp derived from the vector pRS424 (17). The *r2LEU2r2* marker included flanking repeats of 252 bp derived from the vector YEp24 (18). These materials allowed us to carry out the triple-mutant strain construction outlined above.

**FIG 3:**
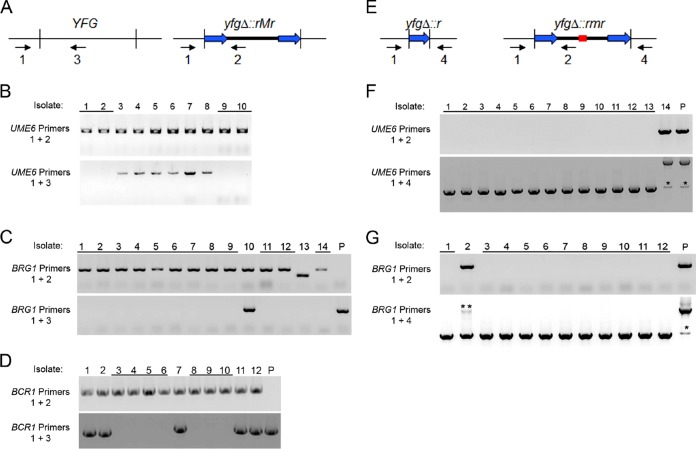
PCR genotype analysis. (A) Primer pairs for detection of deletion alleles. The designation *YFG* refers to any of the genes *UME6*, *BRG1*, or *BCR1*. The designation *yfg*Δ::*rMr* refers to any of the deletion alleles *ume6Δ*::*r1HIS1r1*, *brg1Δ*::*r2LEU2r2*, or *bcr1Δ*::*r1HIS1r1*. Primer 1 anneals to a region flanking the *YFG* gene; primer 2 anneals to a region internal to *yfgΔ*::*rMr* and absent from *YFG*; primer 3 anneals to a region internal to *YFG* and absent from *yfgΔ*::*rMr*. (B) Primer 1, UME6 Check/F; primer 2, HIS1 Check int/R; primer 3, UME6 Check int/R. Genotype assays were performed for 10 His^+^ transformants from transformation 3 with primers for *UME6* alleles. Transformants 1, 2, 9, and 10 yielded PCR products with primers 1 and 2, but not with 1 and 3, as expected for homozygous *ume6Δ*::*r1HIS1r1* mutants. Transformants 4 to 8 yielded PCR products with primers 1 and 2 and with 1 and 3, as expected for heterozygous *UME6/ume6Δ*::*r1HIS1r1* mutants. (C) Primer 1, BRG1 Check/F; primer 2, LEU2 check int/R; primer 3, BRG1 Check int/R. Genotype assays were performed for 14 Leu^+^ His^−^ transformants from transformation 9 with primers for *BRG1* alleles. Transformants 1 to 9, 11, 12, and 14 yielded PCR products with primers 1 and 2 but not with 1 and 3, as expected for homozygous *brg1Δ*::*r2LEU2r2* mutants. Transformant 10 yielded PCR products expected for a heterozygous mutant. Transformant 13 yielded a PCR product indicative of a genetic rearrangement. The parent strain (lane P) was included as a control. (D) Primer 1, BCR1 Check/F; primer 2, HIS1 Check int/R; primer 3, BCR1 Check int/R. Genotype assays were performed for 12 His^+^ Leu^−^ transformants from transformation 13 with primers for *BCR1* alleles. Transformants 3 to 6 and 8 to 10 yielded PCR products as expected for homozygous *bcr1Δ*::*r1HIS1r1* mutants. Transformants 1, 2, 7, 11, and 12 yielded PCR products expected for heterozygous mutants. The parent strain (lane P) was included as a control. (E) Primer pairs for detection of marker loss. The designation yfgΔ::*r* refers to any of the deletion alleles that have lost the selection marker by recombination between repeats, including *ume6Δ*::*r1* or *brg1Δ*::*r2*. The designation *yfg*Δ::*rmr* refers to any of the deletion alleles that have lost a functional marker by a mutation at or near the Cas9-sgRNA cleavage site (represented by the red line segment), which would be designated *ume6Δ*::*r1his1r1* or *brg1Δ*::*r2leu2r2*. Primer 1 anneals to a region flanking the *YFG* gene; primer 2 anneals to a region internal to *yfgΔ*::*rmr*; primer 4 anneals to a region flanking the *YFG* gene on the opposite side from primer 1. (F) Primer 1, UME6 Check/F; primer 2, HIS1 Check int/R; primer 4, UME6 Check down/R. Genotype assays were performed for 14 Leu^+^ His^−^ transformants from transformation 9 with primers for *UME6* alleles. These transformants were the same ones analyzed in panel C. Transformants 1 to 13 yielded PCR products with primers 1 and 4 but not with 1 and 2, as expected for homozygous *ume6Δ*::*r1* mutants. Transformant 14 yielded PCR products expected for a homozygous *ume6Δ*::*r1his1r1* mutant. The parent strain (lane P) was included as a control. The single asterisks mark a minor PCR product that was expected from repeat annealing in the *r1his1r1* and *r1HIS1r1* cassettes (16). (G) Primer 1, BRG1 Check/F; primer 2, LEU2 check int/R; primer 4, BRG1 Check down/R. Genotype assays were performed for 12 His^+^ Leu^−^ transformants from transformation 13 with primers for *BRG1* alleles. These transformants were the same ones analyzed in panel D. Transformants 1 and 3 to 12 yielded PCR products expected for homozygous *brg1Δ*::*r2* mutants. Transformant 2 yielded PCR products expected for a heterozygous *brg1Δ*::*r2/brg1Δ*::*r2leu2r2* mutant. The parent strain (lane P) was included as a control. The single asterisk marks a minor PCR product that was expected from repeat annealing in the *r2leu2r2* and *r2LEU2r2* cassettes (16). The double asterisk marks the PCR product expected for the *brg1Δ*::*r2leu2r2* allele, which was diminished in yield due to the presence of the smaller PCR product from the *brg1Δ*::*r2* allele.

In construction 1, we created a homozygous *ume6Δ*::*r1HIS1r1* mutant (Table 1). All transformations included the gene specifying Cas9. Inclusion of the split-marker template yielded His^+^ transformants (compare transformations 1 and 2 in Table 1). Inclusion of a *UME6-*targeting sgRNA increased the recovery of selected His^+^ transformants considerably (compare transformations 2 and 3 in Table 1), as expected if the Cas9-sgRNA complex were functional. PCR genotyping (Fig. 3A) showed that 4 out of 10 transformants tested were homozygous for the *ume6Δ*::*r1HIS1r1* mutation (Fig. 3B, isolates 1, 2, 9, and 10). This frequency of homozygous marked deletion mutants was similar to what we found previously (10).

**TABLE 1:** Transformation outcomes

Construct	Transformation no.^a^	Recipient strain^b^	Introduced gene			No. of transformants recovered		
			sgRNA1	sgRNA2	Split-marker repair template	Total	His^−^	Leu^−^
*UME6* deletion	1	SN152	0	0	0	0		
	2	SN152	0	0	*ume6Δ*::*r1HIS1r1*	39		
	3	SN152	UME6 (1 µg)	0	*ume6Δ*::*r1HIS1r1*	333		
*BRG1* deletion and *HIS1* excision in *ume6Δ*::*r1HIS1r1*	4	MH101	0	0	0	0		
	5	MH101	0	0	*brg1Δ*::*r2LEU2r2*	12		
	6	MH101	BRG1 (1 µg)	0	*brg1Δ*::*r2LEU2r2*	1,120	0	
	7	MH101	BRG1 (1 µg)	HIS1 (1 µg)	*brg1Δ*::*r2LEU2r2*	564	22	
	8	MH101	BRG1 (1 µg)	HIS1 (3 µg)	*brg1Δ*::*r2LEU2r2*	276	42	
	9	MH101	BRG1 (1 µg)	HIS1 (9 µg)	*brg1Δ*::*r2LEU2r2*	156	30	
*BCR1* deletion and *LEU2* excision in *ume6Δ*::*r1 brg1Δ*::*r2LEU2r2*	10	MH110	0	0	0	0		
	11	MH110	0	0	*bcr1Δ*::*r1HIS1r1*	26		
	12	MH110	BCR1 (1 µg)	0	*bcr1Δ*::*r1HIS1r1*	134		0
	13	MH110	BCR1 (1 µg)	LEU2 (1 µg)	*bcr1Δ*::*r1HIS1r1*	47		22

a All transformations included a *CAS9* gene, following the method of Min et al. (10). Approximate amounts of each PCR product in a typical transformation were the following (unless otherwise stated within the table): *CAS9*, 1 µg; sgRNA1, 1 µg; sgRNA2, 1 µg; split-marker cassette A, 1.5 µg; split marker cassette B, 1.5 µg.

b All strains are of genotype *his1Δ/his1Δ leu2Δ/leu2Δ arg4Δ/arg4Δ*. MH101 has the additional genotype *ume6Δ*::*r1HIS1r1/ume6Δ*::*r1HIS1r1*. MH110 has the additional genotype *ume6Δ*::*r1/ume6Δ*::*r1 brg1Δ*::*r2LEU2r2/brg1Δ*::*r2LEU2r2*, in which the *ume6Δ*::*r1* allele is marked only with one copy of the flanking repeat from the *r1HIS1r1* marker cassette.

In construction 2, we used an *ume6Δ*::*r1HIS1r1* homozygous strain as a recipient to introduce a homozygous *brg1Δ*::*r2LEU2r2* mutation and simultaneously used CRIME to convert the *ume6Δ*::*r1HIS1r1* alleles to *ume6Δ*::*r1* alleles. With a split-marker repair template, inclusion of a *BRG1-*targeting sgRNA increased the recovery of selected Leu^+^ transformants (compare transformations 5 and 6 in Table 1). Inclusion of an additional *HIS1-*targeting sgRNA resulted in 4 to 19% of the Leu^+^ transformants being His^−^, depending upon the amount of the HIS1 sgRNA gene (compare transformations 7 to 9 in Table 1). PCR genotyping (Fig. 3E) indicated that 13 of 14 His^−^ transformants tested were homozygous for a single repeat sequence marking the *ume6Δ*::*r1* alleles (Fig. 3F, isolates 1 to 13). In addition, 12 of 14 His^−^ transformants tested were homozygous for the *brg1Δ*::*r2LEU2r2* mutation (Fig. 3C, isolates 1 to 9, 11, 12, and 14). The results with this construction showed that CRIME allows recycling of the *r1HIS1r1* marker cassette.

In construction 3, we used an *ume6Δ*::*r1 brg1Δ*::*r2LEU2r2* homozygous double-mutant strain as a recipient to introduce a homozygous *bcr1Δ*::*r1HIS1r1* mutation, and we simultaneously used CRIME to convert the *brg1Δ*::*r2LEU2r2* alleles to *brg1Δ*::*r2* alleles. The *r1HIS1r1* cassette we used to select for the *bcr1Δ* allele was the same cassette we used in construction 1 to select for the *ume6Δ* allele. Once again, a split-marker repair template was employed, and inclusion of a *BCR1-*targeting sgRNA increased the recovery of selected His^+^ transformants (compare transformations 11 and 12 in Table 1). Inclusion of an additional *LEU2-*targeting sgRNA resulted in 47% of the His^+^ transformants being Leu^−^ (transformation 13 in Table 1). PCR genotyping indicated that 11 out of 12 Leu^−^ transformants tested were homozygous for a single repeat sequence marking the *brg1Δ*::*r2* alleles (Fig. 3G, isolates 1 and 3 to 12). In addition, 7 out of 12 Leu^−^ transformants tested were homozygous for the *bcr1Δ*::*r1HIS1r1* mutation (Fig. 3D, isolates 3 to 6 and 8 to 10). The results of this construction showed that CRIME allows recycling of the *r2LEU2r2* marker cassette.

## DISCUSSION

We have presented a new approach to marker recycling. Marker recycling has played an important role in genetic manipulation, as illustrated by the hundreds of citations to previous descriptions of marker recycling approaches (1–3). Use of these strategies is especially prominent in fungal studies, where the number of selection markers may be limited (19) and where nutritional requirements can impact diverse phenotypes. Our CRIME approach is conceptually a hybrid between the positive/negative selection strategy and the recombinase-promoted excision strategy. Like the positive/negative selection strategy, CRIME makes use of the cell’s native recombination and repair machinery to excise the DNA between directly repeated sequences. Like the recombinase-promoted excision strategy, CRIME makes use of controlled DNA cleavage events to increase the frequency of recombination in a specific genomic region. CRIME has one major advantage over the prior strategies: speed. This point is illustrated by the fact that each construction in Table 1 required just over 1 week from start to finish, including time for genotyping. In effect, an investigator can use CRIME to steal a little extra time.

The ability of CRISPR-Cas9 systems to be multiplexed is of critical importance for the CRIME strategy. Specifically, Cas9 nuclease subunits can interact with multiple different sgRNAs to target multiple genomic sites for cleavage (9). This multiplexing capability is exploited by CRIME in use of a single transformation for both the deletion of one gene and the recycling of the previously used selection marker. One feature we saw consistently was that inclusion of a second sgRNA reduced the frequency of the transformant class promoted by the first sgRNA (transformations 6 to 9, 12, and 13 in Table 1). These results are expected if there is competition between two sgRNAs or their respective genes. Recognition of the competition phenomenon should prompt investigators to try a range of sgRNA gene concentrations in multiplexed transformations.

Our detailed method employs transformation mixes that contain only PCR products and not cloned DNA segments. The approach builds upon the rapid transient CRISPR-Cas9 approach (10). The use of PCR products saves time compared to cloning-dependent genetic approaches.

The recombinational marker excision event in CRIME seems to be efficient. In our two examples (Fig. 3), 13/14 and 11/12 marker loss events occurred through excision between repeated sequences at both alleles. It is well known that excision between directly repeated regions of homology can be used for double-strand break repair in human cells (20) and yeast cells (21). In the yeast *S. cerevisiae*, the single-strand annealing pathway is operative when repeats are 200 bp or longer; the microhomology-mediated end-joining pathway is used when repeats are 5 to 25 bp in length (21). Thus, the simplest hypothesis is that our CRIME system uses the single-strand annealing pathway. The most important observation in this regard is that small mutations that inactivate the marker gene were rare in our studies. The efficiency of marker loss events from CRIME is important in order to reuse a marker cassette to target a new locus, because extensive homology in the genome might promote integration of the marker at the mutant alleles created previously.

One feature of CRIME that may be viewed as a weakness compared to other marker recycling methods is that two markers are required for CRIME and only one is required for the Ura-blaster and *SAT1* flipper approaches. This consideration will have to be weighed against the time-saving advantage of CRIME in choosing a method to use. A second consideration, more relevant for other fungi than for *C. albicans*, is the relative frequency of marker loss through excision between repeats, as opposed to indels or more complex rearrangements. The first report of CRISPR-Cas9 usage in *Aspergillus fumigatus* presented the startling result that inactivation of a targeted gene was often accompanied by nonhomologous integration of input DNA, in particular the sgRNA gene, at the break site (22). Whether this event would still occur predominantly if flanking repeats were present is unknown. These points illustrate that it is useful to have a few different approaches for any genetic manipulation, because biology and technology often have to reach a compromise when mutations are engineered.

One striking observation was that the efficiency of CRISPR-Cas9-promoted integration seemed to vary widely. Integration of the *brg1Δ* construct was more efficient than that with the *ume6Δ* construct, and both were more efficient than with the *bcr1Δ* construct. These transformations are not precisely comparable though, because they employed different markers and selections, different strains, and different sgRNAs. It may be useful to compare sgRNA efficiencies under parallel conditions, as has been done in human cells (see reference 23 for an example), to see if sgRNA design principles pertinent to *C. albicans* can be deduced.

When we look toward future genetic studies of *C. albicans*, we have a recommendation. Our recommendation is that newly created deletion alleles should be made with repeat-flanked marker cassettes, such as *r1HIS1r1* or *r2LEU2r2*. Many investigators create double- or triple-mutant strains in which deletion mutations are combined to provide an appraisal of pathway relationships or functional redundancy. In the past in our lab, multimutant strain constructions often begin with remaking a single mutant by using a recyclable marker cassette. If most mutant strains in most labs were initially made with recyclable cassettes, then it would be unnecessary to remake mutant strains for genetic interaction studies.

CRISPR-Cas9 systems have been implemented in numerous organisms (9, 22, 24). We suggest that the CRIME approach to marker recycling may be useful in many organisms as well. It relies upon general features of CRISPR-Cas9 systems as well native double-strand break repair machinery, which is highly conserved. Therefore, CRIME seems poised to be applied to diverse genetic systems.

## MATERIALS AND METHODS

### Strains and culture conditions.

All yeast strains are listed in the Table S1 in the supplemental material. Strains were grown at 30°C in YPD plus URI (2% Bacto peptone, 2% dextrose, 1% yeast extract, and 80 µg/ml uridine) with shaking. *C. albicans* transformants were selected on CSM plates lacking either histidine or leucine. All strains were saved as frozen stocks at −80°C in 15% glycerol. All transformations were performed with the lithium acetate transformation method (25) and DNA quantities previously described (10).

10.1128/mSphere.00050-17.1 TABLE S1  Yeast strain genotypes. Download TABLE S1, PDF file, 0.02 MB. Copyright © 2017 Huang and Mitchell. 2017 Huang and Mitchell This content is distributed under the terms of the Creative Commons Attribution 4.0 International license.

### Plasmids and DNA. (i) Overview of partner plasmids for CRIME markers.

**FIG 4:**
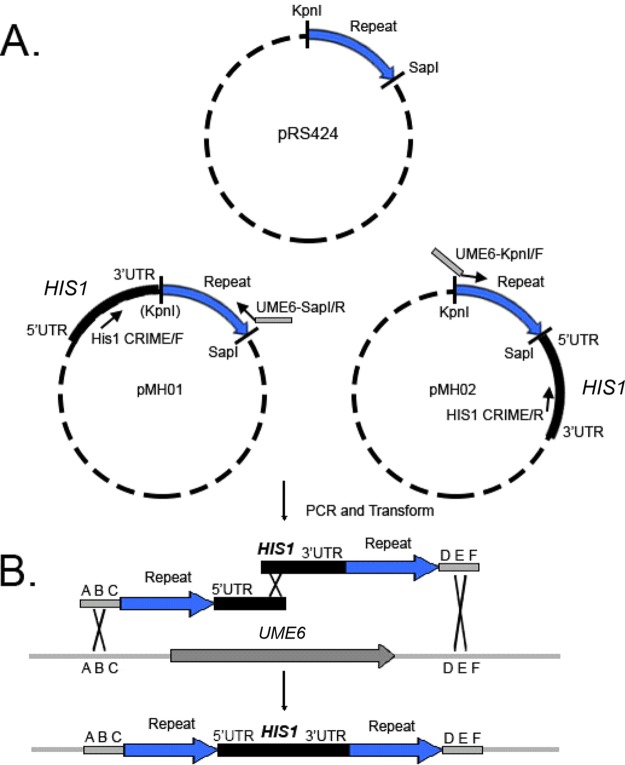
Cloning of Partner plasmids and amplification of the *ume6Δ*::*r1HIS1r1* cassette. (A) pMH01 and pMH02 are derived from pRS424, which contains KpnI and SapI restriction sites. The vector sequence between these restriction sites becomes the repeat sequence for the *ume6Δ*::*r1HIS1r1* cassette. This repeat sequence can be lengthened or shortened through use of different restriction enzymes. (B) Amplification from pMH01 using primers His1 CRIME/F and UME6-SapI/R; the latter primer contained an 80-bp region of homology to the downstream region of UME6 and generated one of the two halves of the *ume6Δ*::*r1HIS1r1* cassette. Amplification from pMH02 using primers His1 CRIME/R and UME6-KpnI/F, the latter primer containing an 80-bp region of homology to the upstream region of UME6, generated the other half of the *ume6Δ*::*r1HIS1r1* cassette. Following transformation, split-marker recombination (16) reconstituted the whole *ume6Δ*::*r1HIS1r1* cassette, revealing the direct repeat.

All primers are listed in the Table S2 in the supplemental material, along with DNA sequences for plasmids pMH01 to -04. We utilized a strategy built around split-marker recombination (16) to generate direct repeat-flanked marker cassettes. Briefly, two plasmids, derived from the same parent, each contain a selectable marker introduced at different restriction sites (Fig. 4A). PCR products that each contain only segments of the whole marker are amplified from these partner plasmids (Fig. 4A). One product contains at its 5′ end an 80-bp region of homology to the upstream region of the gene of interest introduced by a primer. This is followed by the repeat sequence and an incomplete segment of the selectable marker (Fig. 4B). The other product contains at its 5′ end an incomplete segment of the selectable marker and the repeat sequence. This is followed by an 80-bp region of homology to the downstream region of the gene of interest introduced by another primer (Fig. 4B). The two amplicons reconstitute the complete direct repeat-flanked marker *in situ* via split-marker recombination following transformation (Fig. 4B).

10.1128/mSphere.00050-17.2 TABLE S2  Primer sequences. Download TABLE S2, PDF file, 0.02 MB. Copyright © 2017 Huang and Mitchell. 2017 Huang and Mitchell This content is distributed under the terms of the Creative Commons Attribution 4.0 International license.

### (ii) Partner plasmids.

To construct pMH01 and pMH02, each containing the *Candida dubliniensis HIS1* gene, we used the following methods. An aliquot of 1 µg of pRS424 (17) plasmid DNA was digested with the restriction enzyme KpnI, which was then heat inactivated. A second aliquot of pRS424 plasmid DNA was digested with the restriction enzyme SapI, which was then heat inactivated.

To make partner plasmid pMH01 (see Text S1 in the supplemental material for the sequence), a 2.3-kb fragment containing the *Candida dubliniensis HIS1* gene was amplified by PCR from pSN52 (15) using primers KpnI_pRS424_H+AdapN/F and KpnI_pRS424_H+AdapN/R. Four microliters of the PCR product was cotransformed with 1 µl of pRS424 cut with KpnI into *S. cerevisiae* strain BJ8918 (26) to insert the *HIS1* gene into pRS424 at the KpnI restriction site via gap repair (Fig. 4A).

10.1128/mSphere.00050-17.3 TEXT S1  Assembled sequence files for partner plasmids pMH01, pMH02, pMH03, and pMH04. Download TEXT S1, PDF file, 0.03 MB. Copyright © 2017 Huang and Mitchell. 2017 Huang and Mitchell This content is distributed under the terms of the Creative Commons Attribution 4.0 International license.

To make partner plasmid pMH02 (see Text S1), a 2.3-kb fragment containing the *Candida dubliniensis HIS1* gene was amplified by PCR from pSN52 using primers SapI_pRS424_H+AdapN/F and SapI_pRS424_H+AdapN/R. Four microliters of the PCR product was cotransformed with 1 µl of pRS424 cut with SapI into strain BJ8918 to insert the *HIS1* gene into pRS424 at the SapI restriction site via gap repair (Fig. 4A).

Plasmids were recovered from BJ8918 using the Zymoprep yeast plasmid miniprep II kit.

To construct pMH03 and pMH04 (see Text S1 for sequences), each containing the *Candida maltosa LEU2* gene, we used the following methods. An aliquot of YEp24 (18) was digested with the restriction enzyme BamHI, followed by heat inactivation. A second aliquot of pRS424 plasmid DNA was digested with the restriction enzyme SalI, followed by heat inactivation.

To make pMH03, a 2.2-kb fragment containing the *Candida maltosa LEU2* gene was amplified by PCR from pSN40 using primers BamHI_YEp24_H+AdapN/F and BamHI_YEp24_H+AdapN/R. Four microliters of the PCR product was cotransformed with 1 µl of pRS424 cut with BamHI into strain BJ8918 (26) to insert the *LEU2* gene into YEp24 at the BamHI restriction site via gap repair.

To make pMH04, a 2.2-kb fragment containing the *Candida maltosa LEU2* gene was amplified by PCR from pSN40 using primers SalI_YEp24_H+AdapN/F and SalI_YEp24_H+AdapN/R. Four microliters of the PCR product was cotransformed with 1 µl of pRS424 cut with SalI into strain BJ8918 to insert the *LEU2* gene into YEp24 at the SapI restriction site via gap repair.

Plasmids were again recovered from BJ8918 using the Zymoprep yeast plasmid miniprep II kit.

### (iii) CRIME markers.

The *ume6Δ*::*r1HIS1r1* cassette was amplified from pMH01 and pMH02. The aft product was generated by amplification from pMH01 using primers UME6-SapI/R, which contains an 80-bp segment of homology downstream of *UME6*, and HIS1 CRIME/F. The fore product was generated by amplification from pMH02 using primers UME6-KpnI/F, containing 80 bp of homology upstream of *UME6*, and HIS1 CRIME/R.

The *bcr1Δ*::*r1HIS1r1* cassette was amplified from pMH01 and pMH02. The aft product was generated by amplification from pMH01 using primers BCR1-SapI/R, which contains an 80-bp segment of homology downstream of *BCR1*, and UME6-SapI/R. The fore product was generated by amplification from pMH02 using primers BCR1-KpnI/F, containing 80-bp of homology upstream of *BCR1*, and HIS1 CRIME/R.

The *brg1Δ*::*r2LEU2r2* cassette was amplified from pMH03 and pMH04. The aft product was generated by PCR amplification from pMH03 using primers BRG1-SalI/R, containing an 80-bp segment of homology downstream of *BRG1*, and LEU2 CRIME/F. The fore product was generated by PCR amplification from pMH04 using primers BRG1-BamHI/F, containing an 80-bp segment of homology upstream of *BRG1*, and LEU2 CRIME/R.

### (iv) Other DNA cassettes.

The approximately 5-kb CaCas9 cassette containing an *ENO1* promoter, the CaCas9 open reading frame (ORF), and a *CYC1* terminator, was amplified from pV1093 (9) using primers CaCas9/For and CaCas9/Rev. The sgRNA cassettes for *UME6*, *BRG1*, *BCR1*, *C. maltosa HIS1*, and *C. dubliniensis LEU2* were amplified via split-joint PCR as previously described (10) using primer pairs UME6-sgRNA/F and UME6-sgRNA/R, BRG1-sgRNA/F and BRG1-sgRNA/R, BCR1-sgRNA/F and BCR1-sgRNA/R, Cd.HIS1-sgRNA/F and Cd.HIS1-sgRNA/R, and Cm.LEU2-sgRNA/F and Cm.LEU2-sgRNA/R, respectively. The methods, previously described by Min et al. (10), may be summarized as follows: YFG single-guide RNA sequences were first selected, either from the *Candida albicans* CRISPR target sequence database kindly supplied by Vyas et al. (*UME6*, *BRG1*, *BCR1*) (9) or otherwise designed by hand (*C. maltosa HIS1* and *C. dubliniensis LEU2*) (9). The guide sequence was designed into the YFG-sgRNA/F primer sequence by removing the NGG PAM sequence and adding our sgRNA scaffold adapter sequence in its place (i.e., 5′-[YFG target without PAM]-GTTTTAGAGCTAGAAATAGCAAGTTAAA-3’).

The YFG-SNR52/R primer sequence was designed with the reverse complement (i.e., 5′-[reverse complement]-CAAATTAAAAATAGTTTACGCAAGTC-3′). The promoter region was then amplified via PCR with primers SNR52/F and YFG-SNR52/R, while the scaffold and terminator regions were amplified via PCR with primers YFG-sgRNA/F and sgRNA/R. Standard TaKaRa *Ex Taq* protocols were applied for this reaction. Products were then purified using the protocols and materials provided in the Thermo Fisher GeneJet PCR purification kit.

The second round of PCR was roughly modified from the standard TaKaRa *Ex Taq* protocol. To join the *SNR52* promoter amplicon to the sgRNA scaffold and terminator amplicon, equimolar quantities of each amplicon (up to 1,000 ng) were combined roughly as follows: 2.5 µl of purified *SNR52* promoter amplicon, 2.5 µl of purified sgRNA amplicon, 2.5 µl of 10× TaKaRa *Taq* buffer, 2.0 µl of deoxynucleoside triphosphates, and 0.25 µl of TaKaRa *Ex Taq* polymerase, with double-distilled water to a total volume of 25 µl.

The second round of PCR ran for 10 cycles with 30 s at melting temperature, 10 min at 58°C to anneal the two amplicons, and a 5-min elongation phase. One microliter of the second-round PCR product was then amplified in a third round of PCR with primers SNR52/N and sgRNA/N and using standard protocols.
