# Supplementary material for: Marker Recycling in Candida albicans through CRISPR-Cas9-Induced Marker Excision
Source: mSphere. 2017 Mar 15;2(2):e00050-17. doi: 10.1128/mSphere.00050-17 (PMC5352831; doi:10.1128/mSphere.00050-17)
Supplement: TABLE S1 [file sph002172252st1.pdf]

Supplemental Table 1. Yeast Strains

Candida albicans strains:	
SN152	*arg4Δ/arg4Δ leu2Δ/leu2Δ his1Δ/his1Δ URA3/ura3Δ::imm434 IRO1/iro1Δ::imm434*
MH101	*ume6Δ::r1HIS1r1/ume6Δ::r1HIS1r1 arg4Δ/arg4Δ leu2Δ/leu2Δ his1Δ/his1Δ URA3/ura3Δ::imm434 IRO1/iro1Δ::imm434*
MH110	*brg1Δ::r2LEU2r2/brg1Δ::r2LEU2r2 ume6Δ::r/ume6Δ::r arg4Δ/arg4Δ leu2Δ/leu2Δ his1Δ/his1Δ URA3/ura3Δ::imm434 IRO1/iro1Δ::imm434*
Saccharomyces cerevisiae strains:	
BJ8918	*MAT*a *trp1Δ his3-Δ200 ura3-52 leu2Δ*
