# Supplementary material for: Marker Recycling in Candida albicans through CRISPR-Cas9-Induced Marker Excision
Source: mSphere. 2017 Mar 15;2(2):e00050-17. doi: 10.1128/mSphere.00050-17 (PMC5352831; doi:10.1128/mSphere.00050-17)
Supplement: TABLE S2 [file sph002172252st2.pdf]

Supplemental Table 2. Primers

sgRNA Primers:	
BCR1-sgRNA/F	atcaggtgtacttttcaaagGTTTTAGAGCTAGAAATAGCAAGTTAAA
BCR1-SNR52/R	ctttgaaaagtacacctgatCAAATTAAAAATAGTTTACGCAAGTC
BRG1-sgRNA/F	GTACTACAGAGACACCTGAAGTTTTAGAGCTAGAAATAGCAAGTTAAA
BRG1-SNR52/R	TTCAGGTGTCTCTGTAGTACCAAATTAAAAATAGTTTACGCAAGTC
Cd.HIS1-sgRNA/F	taaccattactccaggtagaGTTTTAGAGCTAGAAATAGCAAGTTAAA
Cd.HIS1-SNR52/R	tctacctggagtaatggttaCAAATTAAAAATAGTTTACGCAAGTC
Cm.LEU2-sgRNA/F	atcagaactgcagatttaagGTTTTAGAGCTAGAAATAGCAAGTTAAA
Cm.LEU2-SNR52/R	cttaaatctgcagttctgatCAAATTAAAAATAGTTTACGCAAGTC
UME6-sgRNA/F	GGAGTTGGACTTGGAAGTAGGTTTTAGAGCTAGAAATAGCAAGTTAAA
UME6-SNR52/R	CTACTTCCAAGTCCAACTCCCAAATTAAAAATAGTTTACGCAAGTC
Partner Cloning Primers:	
BCR1_FlankHis_KpnI/F	atattatttaataataacttaaattttcaaaaacaacaactacaacaacta taattaataatactatctcgatatatatcCTCGAGGTCGACGGTATCG
BCR1_FlankHis_SapI/R	aaacatcacaagacataagcaaataaataaataaataaataaaaaatacatt tttcacatactttcttcttattattattgCCAATACGCAAACCGCC
BRG1_FlankLeu_BamHI/F	ATATTTGATATTTCAACGTTATTTCTCCATCCATACTTGTTACATTATAA ATATTCACTACTATTCCAGAAATTCAAATTGACGCATCGTGGCCG
BRG1_FlankLeu_SalI/R	TGTGCAAAAAAATTAAATTAAAACTATTTTAATGACGAATTAAAGGAATTTG GGTTGGGTAAGCAACAGGAATACCGCCAGCGACTCCTGCATTAGGAAGC
HIS1 CRIME/F	gcgcaagaagcctcaact
His1 CRIME/R	gagctacagggcttgacc
LEU2 CRIME/F	tcactttatcgacagttcttctcc
LEU2 CRIME/R	actaactcctgtaacggcg
UME6_FlankHis_KpnI/F	TCATATTCTATATCTTCACTCCAAACATCAATATCCTCAATTTACTTCCAA TTAGTTTTATTTTTTAATCCACTGTATAACTCGAGGTCGACGGTATCG

UME6_FlankHis_SapI/R	ACCAACGGCAACAACAACAACAACAATAACCACCGTCAACCGTCAACCTGT TAATTCTTAATTCTTAACTTAGCCTAATTCCAATACGCAAACCGCC
Primers for Gap Repair of *HIS1* into pRS424	
KpnI_pRS424_H+AdapN/F	AGCGCGCGTAATACGACTCACTATAGGGCGGCCAGTGTGATGGATATCTGCAG
KpnI_pRS424_H+AdapN/R	ATCAAGCTTATCGATACCGTCGACCTCGAGCCGCCAGTGTGCTGGAAT
SapI_pRS424_H+AdapN/F	ACGCGCGGGGAGAGGCGGTTTGCGTATTGGGCCAGTGTGATGGATATCTGCAG
SapI_pRS424_H+AdapN/R	GCAGCCGAACGACCGAGCGCAGCGAGTCAGCCGCCAGTGTGCTGGAAT
Primers for Gap Repair of *LEU2* into YEp24	
BamHI_YEp24_H+AdapN/F	CTATCGACTACGCGATCATGGCGACCACACGCCAGTGTGATGGATATCTGCAG
BamHI_YEp24_H+AdapN/R	TGGCGCCGGTGATGCCGGCCACGATGCGTCCCGCCAGTGTGCTGGAAT
SalI_YEp24_H+AdapN/F	TGGGCTGCTTCCTAATGCAGGAGTCGCATAGCCAGTGTGATGGATATCTGCAG
SalI_YEp24_H+AdapN/R	GGAAGGAGCTGACTGGGTTGAAGGCTCTCACCGCCAGTGTGCTGGAAT
Genotyping Primers	
BCR1 Check Int/R	ACAACCAGGATATCCAGTACA
BCR1 Check/F	GTCTCCTTCAATCAAACGATCA
BRG1 Check Down/R	CGGTTTGATCCAGCTCAGG
BRG1 Check Int/R	GGTATTGATTACTCTTGTCACTGG
BRG1 Check/F	ACGTTATTTCTCCATCCATACTTG
HIS1 Check Int/R	ggctgatttgtctttacatcg
LEU2 Check Int/R	cctcacttgattctgattggc
pRS424 Check/F	GCCATTCGCCATTCAGG
UME6 Check Down/R	GAATCTTCTACGGGAAAAGTTGCAAGA
UME6 Check Int/R	CTATTGGTGTTTGGAAATATGGATTTGC
UME6 Check/F	CATTATTGCTTTGCTTTACATAATTGGTGATAG
Yep24 Check/F	ACAAGGGAGACGCATTGG
