# Supplementary material for: Marker Recycling in Candida albicans through CRISPR-Cas9-Induced Marker Excision
Source: mSphere. 2017 Mar 15;2(2):e00050-17. doi: 10.1128/mSphere.00050-17 (PMC5352831; doi:10.1128/mSphere.00050-17)
Supplement: TEXT S1 [file sph002172252s3.pdf]

>pMH01

TCGCGCGTTTCGGTGATGACGGTGAAAACCTCTGACACATGCAGCTCCCGGAGACGGTCACAGCTTGTCTGTAAGCGGA
TGCCGGGAGCAGACAAGCCCGTCAGGGCGCGTCAGCGGGTGTTGGCGGGTGTCGGGGCTGGCTTAACTATGCGGCATCA
GAGCAGATTGTACTGAGAGTGCACCATAAACGACATTACTATATATATAATATAGGAAGCATTTAATAGACAGCATCGT
AATATATGTGTACTTTGCAGTTATGACGCCAGATGGCAGTAGTGGAAGATATTCTTTATTGAAAAATAGCTTGTCACCT
TACGTACAATCTTGATCCGGAGCTTTTCTTTTTTTGCCGATTAAGAATTAATTCGGTCGAAAAAAGAAAAGGAGAGGGC
CAAGAGGGAGGGCATTGGTGACTATTGAGCACGTGAGTATACGTGATTAAGCACACAAAGGCAGCTTGGAGTATGTCTG
TTATTAATTTCACAGGTAGTTCTGGTCCATTGGTGAAAGTTTGCGGCTTGCAGAGCACAGAGGCCGCAGAATGTGCTCT
AGATTCCGATGCTGACTTGCTGGGTATTATATGTGTGCCCAATAGAAAGAGAACAATTGACCCGGTTATTGCAAGGAAA
ATTTCAAGTCTTGTAAAAGCATATAAAAATAGTTCAGGCACTCCGAAATACTTGGTTGGCGTGTTTCGTAATCAACCTA
AGGAGGATGTTTTGGCTCTGGTCAATGATTACGGCATTGATATCGTCCAACTGCATGGAGATGAGTCGTGGCAAGAATA
CCAAGAGTTCCTCGGTTTGCCAGTTATTAAAAGACTCGTATTTCCAAAAGACTGCAACATACTACTCAGTGCAGCTTCA
CAGAAACCTCATTCGTTTATTCCCTTGTTTGATTCAGAAGCAGGTGGGACAGGTGAACTTTTGGATTGGAACTCGATTT
CTGACTGGGTTGGAAGGCAAGAGAGCCCCGAAAGCTTACATTTTATGTTAGCTGGTGGACTGACGCCAGAAAATGTTGG
TGATGCGCTTAGATTAAATGGCGTTATTGGTGTTGATGTAAGCGGAGGTGTGGAGACAAATGGTGTAAAAGACTCTAAC
AAAATAGCAAATTTCGTCAAAAATGCTAAGAAATAGGTTATTACTGAGTAGTATTTATTTAAGTATTGTTTGTGCACTT
GCCTATGCGGTGTGAAATACCGCACAGATGCGTAAGGAGAAAATACCGCATCAGGAAATTGTAAACGTTAATATTTTGT
TAAAATTCGCGTTAAATTTTTGTTAAATCAGCTCATTTTTTAACCAATAGGCCGAAATCGGCAAAATCCCTTATAAATC
AAAAGAATAGACCGAGATAGGGTTGAGTGTTGTTCCAGTTTGGAACAAGAGTCCACTATTAAAGAACGTGGACTCCAAC
GTCAAAGGGCGAAAAACCGTCTATCAGGGCGATGGCCCACTACGTGAACCATCACCCTAATCAAGTTTTTTGGGGTCGA
GGTGCCGTAAAGCACTAAATCGGAACCCTAAAGGGAGCCCCCGATTTAGAGCTTGACGGGGAAAGCCGGCGAACGTGGC
GAGAAAGGAAGGGAAGAAAGCGAAAGGAGCGGGCGCTAGGGCGCTGGCAAGTGTAGCGGTCACGCTGCGCGTAACCACC
ACACCCGCCGCGCTTAATGCGCCGCTACAGGGCGCGTCGCGCCATTCGCCATTCAGGCTGCGCAACTGTTGGGAAGGGC
GATCGGTGCGGGCCTCTTCGCTATTACGCCAGCTGGCGAAAGGGGGATGTGCTGCAAGGCGATTAAGTTGGGTAACGCC
AGGGTTTTCCCAGTCACGACGTTGTAAAACGACGGCCAGTGAGCGCGCGTAATACGACTCACTATAGGGCGGCCAGTGT
GATGGATATCTGCAGAATTCGCCCTTctagaaaaccgtaccaggtgaactgtttaattggaggatacgatgtaaagaca
aatcagccgtcgttgaattggatagattatttaggtacgcaagttgaacgtccttatggtgctcatggctacgcagcat
tttacacaacatccttgttagataaacattatagaaaggacatgacagttgaagatggtttgaagttaatggacatgtg
tgtcaaagaattacaaacgagaatgcccattgattttaaaggtgtgtacatcaaggtggtagataaagatggtataaga
cagattgagtcaaattgaagtaaatctataagtatgtatatatagtattttattaaactctacaatttgatatctcgag
caccaatatatctgttgcgccagctttcttcaattcatccattacattaccaatttcctttctgttaaccatggaagaa
atggcaacccagtcatcttcgtcgtcactgtgtttatccaaagtggagacagtggctgcccttctacctggagtaatgg
ttaagcactttgattgaatggattttggagcattgtagttacacaagacatattcctgagccgctaaaacaccttgaag
tctttggacaattatgttgaccatttctggaaatttggttttcttggaggaaatcaaatgagcggaagtttccaatatg
gtttcgatcgcctttaatccagcagctttcatggtctctccactttcaactaaatcgacaatggcgtcggcaacaccca
atgcgcaagaagcctcaactgaaccaccaacgtatctaatattcgtaggtttgtcggataatttcttgaaatagtcagt
actcaactttgtaaaggaggatacaatcttctttccaacgagttgttcgggtttttcgtactcgccatttgcaggaact

tggatctgcaatttacatgagccaaacttcaaatccaacaaatcctcaatgttgtcgaattggtctgcttctttgatct
ggtctaacccagttatacccaagtcacaattaccttctccaacgaaaactggaatatcggctgcaggcaaaaagattaa
tgcaattggcaagtttgtggaaagggcaatatccaatctattagatcttctaaactgtatatcggcaccgctcaataag
ctgcagcattttcatataatctgccctttttgggaacagcaaataacaaacgatcgggcaaatgattgactaaatcca
tagtgaatatgtaggtggtgttagaagaaaagtggtatatatttggtgtgggaaaaatcttcaccaaaaaaaaaaaaaa
ataaaaaaatttcaatgactcaatacatttttttttgtacccgttatcgctttgagacacgtttcaaagaaacacctga
aataaacaaggtgtacgtttcaaaaaaaaaaaaaaaaactattgaataaataaaatactgtcctattactttttgcctat
atcttcaacatcttcttcttcggatgtcactgtatcctcttcagttcctaaattgttactactaacttcggtctcttcg
tcctccaggtaccggtcaagccctgtagctccatttggtgtgtttattaattcttcttccatttggctcttcactacca
agttattactaaccccatatccatttgggtttccatacatgtatttgttaaacccccagttgagtaaaatcttgagcat
atcccatggtctagaatgccggccataacttgtcgacaaacacaactgcacaatctggcgacaccgtttgccataaaag
aaagtctgaaagtctaaatgttcctgatgttcttactagtaaatccaatgggggcacatctttcgtataaagatgcttg
tcgatagcctcctcggttatttcaataccacctttcgtcgcttcgtctactactgacttgatcagatgggttatctcgt
ctcttgatgtatatgggaagcagacattcaacacagcacgcttattgtttttagttatttcttcggttctttcaaggat
ctctaacacatctttcggcaaaagtttggtatttcctaatattctgattttaaccccatactcctcacacaacaatcca
tgttggtttatttgcgtaaacttggatttggctaattccatcaaccatttgacctcatattgcagtctcttgaagtttt
cgatcgaaaatgcataaacggtggcacatttcacacccagctcgtataataattctagtatgcttgccatggtatcaaa
cAAGGGCGAATTCCAGCACACTGGCGGCTCGAGGTCGACGGTATCGATAAGCTTGATATCGAATTCCTGCAGCCCGGGG
GATCCACTAGTTCTAGAGCGGCCGCCACCGCGGTGGAGCTCCAGCTTTTGTTCCCTTTAGTGAGGGTTAATTGCGCGCT
TGGCGTAATCATGGTCATAGCTGTTTCCTGTGTGAAATTGTTATCCGCTCACAATTCCACACAACATAGGAGCCGGAAG
CATAAAGTGTAAAGCCTGGGGTGCCTAATGAGTGAGGTAACTCACATTAATTGCGTTGCGCTCACTGCCCGCTTTCCAG
TCGGGAAACCTGTCGTGCCAGCTGCATTAATGAATCGGCCAACGCGCGGGGAGAGGCGGTTTGCGTATTGGGCGCTCTT
CCGCTTCCTCGCTCACTGACTCGCTGCGCTCGGTCGTTCGGCTGCGGCGAGCGGTATCAGCTCACTCAAAGGCGGTAAT
ACGGTTATCCACAGAATCAGGGGATAACGCAGGAAAGAACATGTGAGCAAAAGGCCAGCAAAAGGCCAGGAACCGTAAA
AAGGCCGCGTTGCTGGCGTTTTTCCATAGGCTCCGCCCCCCTGACGAGCATCACAAAAATCGACGCTCAAGTCAGAGGT
GGCGAAACCCGACAGGACTATAAAGATACCAGGCGTTTCCCCCTGGAAGCTCCCTCGTGCGCTCTCCTGTTCCGACCCT
GCCGCTTACCGGATACCTGTCCGCCTTTCTCCCTTCGGGAAGCGTGGCGCTTTCTCATAGCTCACGCTGTAGGTATCTC
AGTTCGGTGTAGGTCGTTCGCTCCAAGCTGGGCTGTGTGCACGAACCCCCCGTTCAGCCCGACCGCTGCGCCTTATCCG
GTAACTATCGTCTTGAGTCCAACCCGGTAAGACACGACTTATCGCCACTGGCAGCAGCCACTGGTAACAGGATTAGCAG
AGCGAGGTATGTAGGCGGTGCTACAGAGTTCTTGAAGTGGTGGCCTAACTACGGCTACACTAGAAGGACAGTATTTGGT
ATCTGCGCTCTGCTGAAGCCAGTTACCTTCGGAAAAAGAGTTGGTAGCTCTTGATCCGGCAAACAAACCACCGCTGGTA
GCGGTGGTTTTTTTGTTTGCAAGCAGCAGATTACGCGCAGAAAAAAAGGATCTCAAGAAGATCCTTTGATCTTTTCTAC
GGGGTCTGACGCTCAGTGGAACGAAAACTCACGTTAAGGGATTTTGGTCATGAGATTATCAAAAAGGATCTTCACCTAG
ATCCTTTTAAATTAAAAATGAAGTTTTAAATCAATCTAAAGTATATATGAGTAAACTTGGTCTGACAGTTACCAATGCT
TAATCAGTGAGGCACCTATCTCAGCGATCTGTCTATTTCGTTCATCCATAGTTGCCTGACTCCCCGTCGTGTAGATAAC
TACGATACGGGAGGGCTTACCATCTGGCCCCAGTGCTGCAATGATACCGCGAGACCCACGCTCACCGGCTCCAGATTTA
TCAGCAATAAACCAGCCAGCCGGAAGGGCCGAGCGCAGAAGTGGTCCTGCAACTTTATCCGCCTCCATCCAGTCTATTA
ATTGTTGCCGGGAAGCTAGAGTAAGTAGTTCGCCAGTTAATAGTTTGCGCAACGTTGTTGCCATTGCTACAGGCATCGT

GGTGTCACGCTCGTCGTTTGGTATGGCTTCATTCAGCTCCGGTTCCCAACGATCAAGGCGAGTTACATGATCCCCCATG
TTGTGCAAAAAAGCGGTTAGCTCCTTCGGTCCTCCGATCGTTGTCAGAAGTAAGTTGGCCGCAGTGTTATCACTCATGG
TTATGGCAGCACTGCATAATTCTCTTACTGTCATGCCATCCGTAAGATGCTTTTCTGTGACTGGTGAGTACTCAACCAA
GTCATTCTGAGAATAGTGTATGCGGCGACCGAGTTGCTCTTGCCCGGCGTCAATACGGGATAATACCGCGCCACATAGC
AGAACTTTAAAAGTGCTCATCATTGGAAAACGTTCTTCGGGGCGAAAACTCTCAAGGATCTTACCGCTGTTGAGATCCA
GTTCGATGTAACCCACTCGTGCACCCAACTGATCTTCAGCATCTTTTACTTTCACCAGCGTTTCTGGGTGAGCAAAAAC
AGGAAGGCAAAATGCCGCAAAAAAGGGAATAAGGGCGACACGGAAATGTTGAATACTCATACTCTTCCTTTTTCAATAT
TATTGAAGCATTTATCAGGGTTATTGTCTCATGAGCGGATACATATTTGAATGTATTTAGAAAAATAAACAAATAGGGG
TTCCGCGCACATTTCCCCGAAAAGTGCCACCTGAACGAAGCATCTGTGCTTCATTTTGTAGAACAAAAATGCAACGCGA
GAGCGCTAATTTTTCAAACAAAGAATCTGAGCTGCATTTTTACAGAACAGAAATGCAACGCGAAAGCGCTATTTTACCA
ACGAAGAATCTGTGCTTCATTTTTGTAAAACAAAAATGCAACGCGAGAGCGCTAATTTTTCAAACAAAGAATCTGAGCT
GCATTTTTACAGAACAGAAATGCAACGCGAGAGCGCTATTTTACCAACAAAGAATCTATACTTCTTTTTTGTTCTACAA
AAATGCATCCCGAGAGCGCTATTTTTCTAACAAAGCATCTTAGATTACTTTTTTTCTCCTTTGTGCGCTCTATAATGCA
GTCTCTTGATAACTTTTTGCACTGTAGGTCCGTTAAGGTTAGAAGAAGGCTACTTTGGTGTCTATTTTCTCTTCCATAA
AAAAAGCCTGACTCCACTTCCCGCGTTTACTGATTACTAGCGAAGCTGCGGGTGCATTTTTTCAAGATAAAGGCATCCC
CGATTATATTCTATACCGATGTGGATTGCGCATACTTTGTGAACAGAAAGTGATAGCGTTGATGATTCTTCATTGGTCA
GAAAATTATGAACGGTTTCTTCTATTTTGTCTCTATATACTACGTATAGGAAATGTTTACATTTTCGTATTGTTTTCGA
TTCACTCTATGAATAGTTCTTACTACAATTTTTTTGTCTAAAGAGTAATACTAGAGATAAACATAAAAAATGTAGAGGT
CGAGTTTAGATGCAAGTTCAAGGAGCGAAAGGTGGATGGGTAGGTTATATAGGGATATAGCACAGAGATATATAGCAAA
GAGATACTTTTGAGCAATGTTTGTGGAAGCGGTATTCGCAATATTTTAGTAGCTCGTTACAGTCCGGTGCGTTTTTGGT
TTTTTGAAAGTGCGTCTTCAGAGCGCTTTTGGTTTTCAAAAGCGCTCTGAAGTTCCTATACTTTCTAGAGAATAGGAAC
TTCGGAATAGGAACTTCAAAGCGTTTCCGAAAACGAGCGCTTCCGAAAATGCAACGCGAGCTGCGCACATACAGCTCAC
TGTTCACGTCGCACCTATATCTGCGTGTTGCCTGTATATATATATACATGAGAAGAACGGCATAGTGCGTGTTTATGCT
TAAATGCGTACTTATATGCGTCTATTTATGTAGGATGAAAGGTAGTCTAGTACCTCCTGTGATATTATCCCATTCCATG
CGGGGTATCGTATGCTTCCTTCAGCACTACCCTTTAGCTGTTCTATATGCTGCCACTCCTCAATTGGATTAGTCTCATC
CTTCAATGCTATCATTTCCTTTGATATTGGATCATATTAAGAAACCATTATTATCATGACATTAACCTATAAAAATAGG
CGTATCACGAGGCCCTTTCGTC

>pMH02

TCGCGCGTTTCGGTGATGACGGTGAAAACCTCTGACACATGCAGCTCCCGGAGACGGTCACAGCTTGTCTGTAAGCGGA
TGCCGGGAGCAGACAAGCCCGTCAGGGCGCGTCAGCGGGTGTTGGCGGGTGTCGGGGCTGGCTTAACTATGCGGCATCA
GAGCAGATTGTACTGAGAGTGCACCATAAACGACATTACTATATATATAATATAGGAAGCATTTAATAGACAGCATCGT
AATATATGTGTACTTTGCAGTTATGACGCCAGATGGCAGTAGTGGAAGATATTCTTTATTGAAAAATAGCTTGTCACCT
TACGTACAATCTTGATCCGGAGCTTTTCTTTTTTTGCCGATTAAGAATTAATTCGGTCGAAAAAAGAAAAGGAGAGGGC
CAAGAGGGAGGGCATTGGTGACTATTGAGCACGTGAGTATACGTGATTAAGCACACAAAGGCAGCTTGGAGTATGTCTG
TTATTAATTTCACAGGTAGTTCTGGTCCATTGGTGAAAGTTTGCGGCTTGCAGAGCACAGAGGCCGCAGAATGTGCTCT
AGATTCCGATGCTGACTTGCTGGGTATTATATGTGTGCCCAATAGAAAGAGAACAATTGACCCGGTTATTGCAAGGAAA
ATTTCAAGTCTTGTAAAAGCATATAAAAATAGTTCAGGCACTCCGAAATACTTGGTTGGCGTGTTTCGTAATCAACCTA
AGGAGGATGTTTTGGCTCTGGTCAATGATTACGGCATTGATATCGTCCAACTGCATGGAGATGAGTCGTGGCAAGAATA
CCAAGAGTTCCTCGGTTTGCCAGTTATTAAAAGACTCGTATTTCCAAAAGACTGCAACATACTACTCAGTGCAGCTTCA
CAGAAACCTCATTCGTTTATTCCCTTGTTTGATTCAGAAGCAGGTGGGACAGGTGAACTTTTGGATTGGAACTCGATTT
CTGACTGGGTTGGAAGGCAAGAGAGCCCCGAAAGCTTACATTTTATGTTAGCTGGTGGACTGACGCCAGAAAATGTTGG
TGATGCGCTTAGATTAAATGGCGTTATTGGTGTTGATGTAAGCGGAGGTGTGGAGACAAATGGTGTAAAAGACTCTAAC
AAAATAGCAAATTTCGTCAAAAATGCTAAGAAATAGGTTATTACTGAGTAGTATTTATTTAAGTATTGTTTGTGCACTT
GCCTATGCGGTGTGAAATACCGCACAGATGCGTAAGGAGAAAATACCGCATCAGGAAATTGTAAACGTTAATATTTTGT
TAAAATTCGCGTTAAATTTTTGTTAAATCAGCTCATTTTTTAACCAATAGGCCGAAATCGGCAAAATCCCTTATAAATC
AAAAGAATAGACCGAGATAGGGTTGAGTGTTGTTCCAGTTTGGAACAAGAGTCCACTATTAAAGAACGTGGACTCCAAC
GTCAAAGGGCGAAAAACCGTCTATCAGGGCGATGGCCCACTACGTGAACCATCACCCTAATCAAGTTTTTTGGGGTCGA
GGTGCCGTAAAGCACTAAATCGGAACCCTAAAGGGAGCCCCCGATTTAGAGCTTGACGGGGAAAGCCGGCGAACGTGGC
GAGAAAGGAAGGGAAGAAAGCGAAAGGAGCGGGCGCTAGGGCGCTGGCAAGTGTAGCGGTCACGCTGCGCGTAACCACC
ACACCCGCCGCGCTTAATGCGCCGCTACAGGGCGCGTCGCGCCATTCGCCATTCAGGCTGCGCAACTGTTGGGAAGGGC
GATCGGTGCGGGCCTCTTCGCTATTACGCCAGCTGGCGAAAGGGGGATGTGCTGCAAGGCGATTAAGTTGGGTAACGCC
AGGGTTTTCCCAGTCACGACGTTGTAAAACGACGGCCAGTGAGCGCGCGTAATACGACTCACTATAGGGCGAATTGGGT
ACCGGGCCCCCCCTCGAGGTCGACGGTATCGATAAGCTTGATATCGAATTCCTGCAGCCCGGGGGATCCACTAGTTCTA
GAGCGGCCGCCACCGCGGTGGAGCTCCAGCTTTTGTTCCCTTTAGTGAGGGTTAATTGCGCGCTTGGCGTAATCATGGT
CATAGCTGTTTCCTGTGTGAAATTGTTATCCGCTCACAATTCCACACAACATAGGAGCCGGAAGCATAAAGTGTAAAGC
CTGGGGTGCCTAATGAGTGAGGTAACTCACATTAATTGCGTTGCGCTCACTGCCCGCTTTCCAGTCGGGAAACCTGTCG
TGCCAGCTGCATTAATGAATCGGCCAACGCGCGGGGAGAGGCGGTTTGCGTATTGGGCCAGTGTGATGGATATCTGCAG
AATTCGCCCTTctagaaaaccgtaccaggtgaactgtttaattggaggatacgatgtaaagacaaatcagccgtcgttg
aattggatagattatttaggtacgcaagttgaacgtccttatggtgctcatggctacgcagcattttacacaacatcct
tgttagataaacattatagaaaggacatgacagttgaagatggtttgaagttaatggacatgtgtgtcaaagaattaca
aacgagaatgcccattgattttaaaggtgtgtacatcaaggtggtagataaagatggtataagacagattgagtcaaat
tgaagtaaatctataagtatgtatatatagtattttattaaactctacaatttgatatctcgagcaccaatatatctgt
tgcgccagctttcttcaattcatccattacattaccaatttcctttctgttaaccatggaagaaatggcaacccagtca
tcttcgtcgtcactgtgtttatccaaagtggagacagtggctgcccttctacctggagtaatggttaagcactttgatt

gaatggattttggagcattgtagttacacaagacatattcctgagccgctaaaacaccttgaagtctttggacaattat
gttgaccatttctggaaatttggttttcttggaggaaatcaaatgagcggaagtttccaatatggtttcgatcgccttt
aatccagcagctttcatggtctctccactttcaactaaatcgacaatggcgtcggcaacacccaatgcgcaagaagcct
caactgaaccaccaacgtatctaatattcgtaggtttgtcggataatttcttgaaatagtcagtactcaactttgtaaa
ggaggatacaatcttctttccaacgagttgttcgggtttttcgtactcgccatttgcaggaacttggatctgcaattta
catgagccaaacttcaaatccaacaaatcctcaatgttgtcgaattggtctgcttctttgatctggtctaacccagtta
tacccaagtcacaattaccttctccaacgaaaactggaatatcggctgcaggcaaaaagattaatgcaattggcaagtt
tgtggaaagggcaatatccaatctattagatcttctaaactgtatatcggcaccgctcaataagctgcagcatttttca
tataatctgcccttttttgggaacagcaaataacaaacgatcgggcaaatgattgactaaatccatagtgaatatgtagg
tggtgttagaagaaaagtggtatatatttggtgtgggaaaaatcttcaccaaaaaaaaaaaaaaataaaaaaatttcaa
tgactcaatacatttttttttgtacccgttatcgctttgagacacgtttcaaagaaacacctgaaataaacaaggtgta
cgtttcaaaaaaaaaaaaaaaactattgaataaataaaatactgtcctattactttttgcctatatcttcaacatcttc
ttcttcggatgtcactgtatcctcttcagttcctaaattgttactactaacttcggtctcttcgtcctccaggtaccgg
tcaagccctgtagctccatttggtgtgtttattaattcttcttccatttggctcttcactaccaagttattactaaccc
catatccatttgggtttccatacatgtatttgttaaacccccagttgagtaaaatcttgagcatatcccatggtctaga
atgccggccataacttgtcgacaaacacaactgcacaatctggcgacaccgtttgccataaaagaaagtctgaaagtct
aaatgttcctgatgttcttactagtaaatccaatgggggcacatctttcgtataaagatgcttgtcgatagcctcctcg
gttatttcaataccacctttcgtcgcttcgtctactactgacttgatcagatgggttatctcgtctcttgatgtatatg
ggaagcagacattcaacacagcacgcttattgtttttagttatttcttcggttctttcaaggatctctaacacatcttt
cggcaaaagtttggtatttcctaatattctgattttaaccccatactcctcacacaacaatccatgttggtttatttgc
gtaaacttggatttggctaattccatcaaccatttgacctcatattgcagtctcttgaagttttcgatcgaaaatgcat
aaacggtggcacatttcacacccagctcgtataataattctagtatgcttgccatggtatcaaacAAGGGCGAATTCCA
GCACACTGGCGGCTGACTCGCTGCGCTCGGTCGTTCGGCTGCGGCGAGCGGTATCAGCTCACTCAAAGGCGGTAATACG
GTTATCCACAGAATCAGGGGATAACGCAGGAAAGAACATGTGAGCAAAAGGCCAGCAAAAGGCCAGGAACCGTAAAAAG
GCCGCGTTGCTGGCGTTTTTCCATAGGCTCCGCCCCCCTGACGAGCATCACAAAAATCGACGCTCAAGTCAGAGGTGGC
GAAACCCGACAGGACTATAAAGATACCAGGCGTTTCCCCCTGGAAGCTCCCTCGTGCGCTCTCCTGTTCCGACCCTGCC
GCTTACCGGATACCTGTCCGCCTTTCTCCCTTCGGGAAGCGTGGCGCTTTCTCATAGCTCACGCTGTAGGTATCTCAGT
TCGGTGTAGGTCGTTCGCTCCAAGCTGGGCTGTGTGCACGAACCCCCCGTTCAGCCCGACCGCTGCGCCTTATCCGGTA
ACTATCGTCTTGAGTCCAACCCGGTAAGACACGACTTATCGCCACTGGCAGCAGCCACTGGTAACAGGATTAGCAGAGC
GAGGTATGTAGGCGGTGCTACAGAGTTCTTGAAGTGGTGGCCTAACTACGGCTACACTAGAAGGACAGTATTTGGTATC
TGCGCTCTGCTGAAGCCAGTTACCTTCGGAAAAAGAGTTGGTAGCTCTTGATCCGGCAAACAAACCACCGCTGGTAGCG
GTGGTTTTTTTGTTTGCAAGCAGCAGATTACGCGCAGAAAAAAAGGATCTCAAGAAGATCCTTTGATCTTTTCTACGGG
GTCTGACGCTCAGTGGAACGAAAACTCACGTTAAGGGATTTTGGTCATGAGATTATCAAAAAGGATCTTCACCTAGATC
CTTTTAAATTAAAAATGAAGTTTTAAATCAATCTAAAGTATATATGAGTAAACTTGGTCTGACAGTTACCAATGCTTAA
TCAGTGAGGCACCTATCTCAGCGATCTGTCTATTTCGTTCATCCATAGTTGCCTGACTCCCCGTCGTGTAGATAACTAC
GATACGGGAGGGCTTACCATCTGGCCCCAGTGCTGCAATGATACCGCGAGACCCACGCTCACCGGCTCCAGATTTATCA
GCAATAAACCAGCCAGCCGGAAGGGCCGAGCGCAGAAGTGGTCCTGCAACTTTATCCGCCTCCATCCAGTCTATTAATT
GTTGCCGGGAAGCTAGAGTAAGTAGTTCGCCAGTTAATAGTTTGCGCAACGTTGTTGCCATTGCTACAGGCATCGTGGT

GTCACGCTCGTCGTTTGGTATGGCTTCATTCAGCTCCGGTTCCCAACGATCAAGGCGAGTTACATGATCCCCCATGTTG
TGCAAAAAAGCGGTTAGCTCCTTCGGTCCTCCGATCGTTGTCAGAAGTAAGTTGGCCGCAGTGTTATCACTCATGGTTA
TGGCAGCACTGCATAATTCTCTTACTGTCATGCCATCCGTAAGATGCTTTTCTGTGACTGGTGAGTACTCAACCAAGTC
ATTCTGAGAATAGTGTATGCGGCGACCGAGTTGCTCTTGCCCGGCGTCAATACGGGATAATACCGCGCCACATAGCAGA
ACTTTAAAAGTGCTCATCATTGGAAAACGTTCTTCGGGGCGAAAACTCTCAAGGATCTTACCGCTGTTGAGATCCAGTT
CGATGTAACCCACTCGTGCACCCAACTGATCTTCAGCATCTTTTACTTTCACCAGCGTTTCTGGGTGAGCAAAAACAGG
AAGGCAAAATGCCGCAAAAAAGGGAATAAGGGCGACACGGAAATGTTGAATACTCATACTCTTCCTTTTTCAATATTAT
TGAAGCATTTATCAGGGTTATTGTCTCATGAGCGGATACATATTTGAATGTATTTAGAAAAATAAACAAATAGGGGTTC
CGCGCACATTTCCCCGAAAAGTGCCACCTGAACGAAGCATCTGTGCTTCATTTTGTAGAACAAAAATGCAACGCGAGAG
CGCTAATTTTTCAAACAAAGAATCTGAGCTGCATTTTTACAGAACAGAAATGCAACGCGAAAGCGCTATTTTACCAACG
AAGAATCTGTGCTTCATTTTTGTAAAACAAAAATGCAACGCGAGAGCGCTAATTTTTCAAACAAAGAATCTGAGCTGCA
TTTTTACAGAACAGAAATGCAACGCGAGAGCGCTATTTTACCAACAAAGAATCTATACTTCTTTTTTGTTCTACAAAAA
TGCATCCCGAGAGCGCTATTTTTCTAACAAAGCATCTTAGATTACTTTTTTTCTCCTTTGTGCGCTCTATAATGCAGTC
TCTTGATAACTTTTTGCACTGTAGGTCCGTTAAGGTTAGAAGAAGGCTACTTTGGTGTCTATTTTCTCTTCCATAAAAA
AAGCCTGACTCCACTTCCCGCGTTTACTGATTACTAGCGAAGCTGCGGGTGCATTTTTTCAAGATAAAGGCATCCCCGA
TTATATTCTATACCGATGTGGATTGCGCATACTTTGTGAACAGAAAGTGATAGCGTTGATGATTCTTCATTGGTCAGAA
AATTATGAACGGTTTCTTCTATTTTGTCTCTATATACTACGTATAGGAAATGTTTACATTTTCGTATTGTTTTCGATTC
ACTCTATGAATAGTTCTTACTACAATTTTTTTGTCTAAAGAGTAATACTAGAGATAAACATAAAAAATGTAGAGGTCGA
GTTTAGATGCAAGTTCAAGGAGCGAAAGGTGGATGGGTAGGTTATATAGGGATATAGCACAGAGATATATAGCAAAGAG
ATACTTTTGAGCAATGTTTGTGGAAGCGGTATTCGCAATATTTTAGTAGCTCGTTACAGTCCGGTGCGTTTTTGGTTTT
TTGAAAGTGCGTCTTCAGAGCGCTTTTGGTTTTCAAAAGCGCTCTGAAGTTCCTATACTTTCTAGAGAATAGGAACTTC
GGAATAGGAACTTCAAAGCGTTTCCGAAAACGAGCGCTTCCGAAAATGCAACGCGAGCTGCGCACATACAGCTCACTGT
TCACGTCGCACCTATATCTGCGTGTTGCCTGTATATATATATACATGAGAAGAACGGCATAGTGCGTGTTTATGCTTAA
ATGCGTACTTATATGCGTCTATTTATGTAGGATGAAAGGTAGTCTAGTACCTCCTGTGATATTATCCCATTCCATGCGG
GGTATCGTATGCTTCCTTCAGCACTACCCTTTAGCTGTTCTATATGCTGCCACTCCTCAATTGGATTAGTCTCATCCTT
CAATGCTATCATTTCCTTTGATATTGGATCATATTAAGAAACCATTATTATCATGACATTAACCTATAAAAATAGGCGT
ATCACGAGGCCCTTTCGTC

>pMH03

GAATTCTGAACCAGTCCTAAAACGAGTAAATAGGACCGGCAATTCTTCAAGCAATAAACAGGAATACCAATTATTAAAA
GATAACTTAGTCAGATCGTACAATAAAGCTTTGAAGAAAAATGCGCCTTATTCAATCTTTGCTATAAAAAATGGCCCAA
AATCTCACATTGGAAGACATTTGATGACCTCATTTCTTTCAATGAAGGGCCTAACGGAGTTGACTAATGTTGTGGGAAA
TTGGAGCGATAAGCGTGCTTCTGCCGTGGCCAGGACAACGTATACTCATCAGATAACAGCAATACCTGATCACTACTTC
GCACTAGTTTCTCGGTACTATGCATATGATCCAATATCAAAGGAAATGATAGCATTGAAGGATGAGACTAATCCAATTG
AGGAGTGGCAGCATATAGAACAGCTAAAGGGTAGTGCTGAAGGAAGCATACGATACCCCGCATGGAATGGGATAATATC
ACAGGAGGTACTAGACTACCTTTCATCCTACATAAATAGACGCATATAAGTACGCATTTAAGCATAAACACGCACTATG
CCGTTCTTCTCATGTATATATATATACAGGCAACACGCAGATATAGGTGCGACGTGAACAGTGAGCTGTATGTGCGCAG
CTCGCGTTGCATTTTCGGAAGCGCTCGTTTTCGGAAACGCTTTGAAGTTCCTATTCCGAAGTTCCTATTCTCTAGAAAG
TATAGGAACTTCAGAGCGCTTTTGAAAACCAAAAGCGCTCTGAAGACGCACTTTCAAAAAACCAAAAACGCACCGGACT
GTAACGAGCTACTAAAATATTGCGAATACCGCTTCCACAAACATTGCTCAAAAGTATCTCTTTGCTATATATCTCTGTG
CTATATCCCTATATAACCTACCCATCCACCTTTCGCTCCTTGAACTTGCATCTAAACTCGACCTCTACATTTTTTATGT
TTATCTCTAGTATTACTCTTTAGACAAAAAAATTGTAGTAAGAACTATTCATAGAGTGAATCGAAAACAATACGAAAAT
GTAAACATTTCCTATACGTAGTATATAGAGACAAAATAGAAGAAACCGTTCATAATTTTCTGACCAATGAAGAATCATC
AACGCTATCACTTTCTGTTCACAAAGTATGCGCAATCCACATCGGTATAGAATATAATCGGGGATGCCTTTATCTTGAA
AAAATGCACCCGCAGCTTCGCTAGTAATCAGTAAACGCGGGAAGTGGAGTCAGGCTTTTTTTATGGAAGAGAAAATAGA
CACCAAAGTAGCCTTCTTCTAACCTTAACGGACCTACAGTGCAAAAAGTTATCAAGAGACTGCATTATAGAGCGCACAA
AGGAGAAAAAAAGTAATCTAAGATGCTTTGTTAGAAAAATAGCGCTCTCGGGATGCATTTTTGTAGAACAAAAAAGAAG
TATAGATTCTTTGTTGGTAAAATAGCGCTCTCGCGTTGCATTTCTGTTCTGTAAAAATGCAGCTCAGATTCTTTGTTTG
AAAAATTAGCGCTCTCGCGTTGCATTTTTGTTTTACAAAAATGAAGCACAGATTCTTCGTTGGTAAAATAGCGCTTTCG
CGTTGCATTTCTGTTCTGTAAAAATGCAGCTCAGATTCTTTGTTTGAAAAATTAGCGCTCTCGCGTTGCATTTTTGTTC
TACAAAATGAAGCACAGATGCTTCGTTAACAAAGATATGCTATTGAAGTGCAAGATGGAAACGCAGAAAATGAACCGGG
GATGCGACGTGCAAGATTACCTATGCAATAGATGCAATAGTTTCTCCAGGAACCGAAATACATACATTGTCTTCCGTAA
AGCGCTAGACTATATATTATTATACAGGTTCAAATATACTATCTGTTTCAGGGAAAACTCCCAGGTTCGGATGTTCAAA
ATTCAATGATGGGTAACAAGTACGATCGTAAATCTGTAAAACAGTTTGTCGGATATTAGGCTGTATCTCCTCAAAGCGT
ATTCGAATATCATTGAGAAGCTGCAGCGTCACATCGGATAATAATGATGGCAGCCATTGTAGAAGTGCCTTTTGCATTT
CTAGTCTCTTTCTCGGTCTAGCTAGTTTTACTACATCGCGAAGATAGAATCTTAGATCACACTGCCTTTGCTGAGCTGG
ATCAATAGAGTAACAAAAGAGTGGTAAGGCCTCGTTAAAGGACAAGGACCTGAGCGGAAGTGTATCGTACAGTAGACGG
AGTATACTAGTATAGTCTATAGTCCGTGGAATTCTCATGTTTGACAGCTTATCATCGATAAGCTTTTCAATTCAATTCA
TCATTTTTTTTTTTATTCTTTTTTTTTGATTTCGGTTTCTTTGAAATTTTTTTGATTCGGTAATCTCCGAACAGAAGGAAG
AACGAAGGAAGGAGCACAGACTTAGATTGGTATATATACGCATATGTAGTGTTGAAGAAACATGAAATTGCCCAGTATT
CTTAACCCAACTGCACAGAACAAAAACCTGCAGGAAACGAAGATAAATCATGTCGAAAGCTACATATAAGGAACGTGCT
GCTACTCATCCTAGTCCTGTTGCTGCCAAGCTATTTAATATCATGCACGAAAAGCAAACAAACTTGTGTGCTTCATTGG
ATGTTCGTACCACCAAGGAATTACTGGAGTTAGTTGAAGCATTAGGTCCCAAAATTTGTTTACTAAAAACACATGTGGA
TATCTTGACTGATTTTTCCATGGAGGGCACAGTTAAGCCGCTAAAGGCATTATCCGCCAAGTACAATTTTTTACTCTTC
GAAGACAGAAAATTTGCTGACATTGGTAATACAGTCAAATTGCAGTACTCTGCGGGTGTATACAGAATAGCAGAATGGG

CAGACATTACGAATGCACACGGTGTGGTGGGCCCAGGTATTGTTAGCGGTTTGAAGCAGGCGGCAGAAGAAGTAACAAA
GGAACCTAGAGGCCTTTTGATGTTAGCAGAATTGTCATGCAAGGGCTCCCTATCTACTGGAGAATATACTAAGGGTACT
GTTGACATTGCGAAGAGCGACAAAGATTTTGTTATCGGCTTTATTGCTCAAAGAGACATGGGTGGAAGAGATGAAGGTT
ACGATTGGTTGATTATGACACCCGGTGTGGGTTTAGATGACAAGGGAGACGCATTGGGTCAACAGTATAGAACCGTGGA
TGATGTGGTCTCTACAGGATCTGACATTATTATTGTTGGAAGAGGACTATTTGCAAAGGGAAGGGATGCTAAGGTAGAG
GGTGAACGTTACAGAAAAGCAGGCTGGGAAGCATATTTGAGAAGATGCGGCCAGCAAAACTAAAAAACTGTATTATAAG
TAAATGCATGTATACTAAACTCACAAATTAGAGCTTCAATTTAATTATATCAGTTATTACCCGGGAATCTCGGTCGTAA
TGATTTTTATAATGACGAAAAAAAAAAAAATTGGAAAGAAAAAGCTTTAATGCGGTAGTTTATCACAGTTAAATTGCTAA
CGCAGTCAGGCACCGTGTATGAAATCTAACAATGCGCTCATCGTCATCCTCGGCACCGTCACCCTGGATGCTGTAGGCA
TAGGCTTGGTTATGCCGGTACTGCCGGGCCTCTTGCGGGATATCGTCCATTCCGACAGCATCGCCAGTCACTATGGCGT
GCTGCTAGCGCTATATGCGTTGATGCAATTTCTATGCGCACCCGTTCTCGGAGCACTGTCCGACCGCTTTGGCCGCCGC
CCAGTCCTGCTCGCTTCGCTACTTGGAGCCACTATCGACTACGCGATCATGGCGACCACACCCGCCAGTGTGCTGGAAT
TCGCCCTTggatccaatcatcactggtgggggtcaagaaagaggtttgagatcaggtactttggcaccaccattagtag
ctgggtttggtgaagctgcaagattaatgaaacaagaatctgcatttgacaagaaacatattgagaggttatctactaa
attgaagaacggtttgttatccattccatctactcagtttaacggttgtaataacccaacatatcaatacccaggttgt
gtgaatgtttcctttgcatacattgaaggggaatctttgttaatggcattgaaagatattgcattgagttctggttctg
catgtacctctgcatctttggaaccatcgtatgttttacatgctttgggtgccgatgatgccttggctcattcttccat
tagattcggtattggtagattcaccactgaagcagaagttgactatgttattcaagcaattaatgaaagagttgatttc
ttgagaaagatgtctccattatgggaaatggttcaagaaggtattgacttgaactctatcgaatggagtggacattaag
gtttttgtttttttgtaatttgtgttgttatgtgtaattatcgtgtatagagtaatttcggaaattgtactttcgttc
atgtatctgaaccagatgaataatacgttcgtattgttgaagaatgactaactcctgtaacggcgtgcgtgggttccgc
agccgggttcaattttttttttcttgttcactataaaaatatataataaacgaaaaaaatccgagggactcaggctatg
aaatttttcactcaagaattttttttccttcatccttaattcctttattcactacccaaacccacaatgtcagttaaaa
ctaaaactattacaattttaccaggtgatcacgtcggtaccgaaattgtcaatgaagctatcaaagttttagaagcaat
tgaagctgccactccttaccaaaaaatccatttcgatttcaaacatcatttaatcggtggtgctgccattgatgccact
ggtgtcccattgccagatgacgcacttgaaagtgccaaaaaatctgatgccgtcttattgggtgctgttggtggaccaa
aatgggggactggcgccgttcgtccagaacaaggtttattaaagattcgtaaagaattgaacctttatgctaacatcag
accatgtaactttgccagtgactccttattggaattatccccattgagaccagaagtcgttaaaggtacaaacttgatc
attgttcgtgaattagttggtggtatttatttcggtgatcgtgaagaacaagaagaaagtgaagataaacaaaccgcat
gggatactgaaaaatacaccgtcgatgaagtcaccagaatcacccgtatggctgccttcatggctttacaacataaccc
accattaccaatctggtcattggataaggctaatgttttagcttcttctagattatggagaagaactgtcgataaagtg
atttctgaagagttcccaactttgtctgttcaacatcaattgattgactccgccgccatgattttaattcaaaacccaa
ccaaattgaatggtataatcatcacttctaacatgtttggtgatatcatttccgatgaagcttcagttattccaggttc
cttgggtttattaccatctgcatctttggcttcattgccagataccaacaccgcttttggtctttatgaaccatgtcac
ggttctgctcctgatttaccagccaataaagttaacccaattgctactattttatctgctgcttctatgttgagattat
ctttggattgtgtgaaagaagctgaagctttagaagaagccgtgaaacaagtcttggataaaggtatcagaactgcaga
tttaagaggtaccagttcagctactgaagttggtgacgcgattgtcgaagctgttactaaaatcttaaaagaaaaagct
taagtaaagtatacacaaaatttaacgaatattattaatacaaaatgaacaccacctaaatactttttttttaaaaatgt

accgactcttctaaaaattgcgataattcaatatcggaaatttctctcaccggtttacttggatcttcggaaataaatc
tatttgaagtaatggtagcctcacttgattctgattggctctttctagacatgggtaggtAAGGGCGAATTCTGCAGAT
ATCCATCACACTGGCGACGCATCGTGGCCGGCATCACCGGCGCCACAGGTGCGGTTGCTGGCGCCTATATCGCCGACAT
CACCGATGGGGAAGATCGGGCTCGCCACTTCGGGCTCATGAGCGCTTGTTTCGGCGTGGGTATGGTGGCAGGCCCCGTG
GCCGGGGGACTGTTGGGCGCCATCTCCTTGCATGCACCATTCCTTGCGGCGGCGGTGCTCAACGGCCTCAACCTACTAC
TGGGCTGCTTCCTAATGCAGGAGTCGCATAAGGGAGAGCGTCGACCGATGCCCTTGAGAGCCTTCAACCCAGTCAGCTC
CTTCCGGTGGGCGCGGGGCATGACTATCGTCGCCGCACTTATGACTGTCTTCTTTATCATGCAACTCGTAGGACAGGTG
CCGGCAGCGCTCTGGGTCATTTTCGGCGAGGACCGCTTTCGCTGGAGCGCGACGATGATCGGCCTGTCGCTTGCGGTAT
TCGGAATCTTGCACGCCCTCGCTCAAGCCTTCGTCACTGGTCCCGCCACCAAACGTTTCGGCGAGAAGCAGGCCATTAT
CGCCGGCATGGCGGCCGACGCGCTGGGCTACGTCTTGCTGGCGTTCGCGACGCGAGGCTGGATGGCCTTCCCCATTATG
ATTCTTCTCGCTTCCGGCGGCATCGGGATGCCCGCGTTGCAGGCCATGCTGTCCAGGCAGGTAGATGACGACCATCAGG
GACAGCTTCAAGGATCGCTCGCGGCTCTTACCAGCCTAACTTCGATCACTGGACCGCTGATCGTCACGGCGATTTATGC
CGCCTCGGCGAGCACATGGAACGGGTTGGCATGGATTGTAGGCGCCGCCCTATACCTTGTCTGCCTCCCCGCGTTGCGT
CGCGGTGCATGGAGCCGGGCCACCTCGACCTGAATGGAAGCCGGCGGCACCTCGCTAACGGATTCACCACTCCAAGAAT
TGGAGCCAATCAATTCTTGCGGAGAACTGTGAATGCGCAAACCAACCCTTGGCAGAACATATCCATCGCGTCCGCCATC
TCCAGCAGCCGCACGCGGCGCATCTCGGGCAGCGTTGGGTCCTGGCCACGGGTGCGCATGATCGTGCTCCTGTCGTTGA
GGACCCGGCTAGGCTGGCGGGGTTGCCTTACTGGTTAGCAGAATGAATCACCGATACGCGAGCGAACGTGAAGCGACTG
CTGCTGCAAAACGTCTGCGACCTGAGCAACAACATGAATGGTCTTCGGTTTCCGTGTTTCGTAAAGTCTGGAAACGCGG
AAGTCAGCGCCCTGCACCATTATGTTCCGGATCTGCATCGCAGGATGCTGCTGGCTACCCTGTGGAACACCTACATCTG
TATTAACGAAGCGCTGGCATTGACCCTGAGTGATTTTTCTCTGGTCCCGCCGCATCCATACCGCCAGTTGTTTACCCTC
ACAACGTTCCAGTAACCGGGCATGTTCATCATCAGTAACCCGTATCGTGAGCATCCTCTCTCGTTTCATCGGTATCATT
ACCCCCATGAACAGAAATTCCCCCTTACACGGAGGCATCAAGTGACCAAACAGGAAAAAACCGCCCTTAACATGGCCCG
CTTTATCAGAAGCCAGACATTAACGCTTCTGGAGAAACTCAACGAGCTGGACGCGGATGAACAGGCAGACATCTGTGAA
TCGCTTCACGACCACGCTGATGAGCTTTACCGCAGCTGCCTCGCGCGTTTCGGTGATGACGGTGAAAACCTCTGACACA
TGCAGCTCCCGGAGACGGTCACAGCTTGTCTGTAAGCGGATGCCGGGAGCAGACAAGCCCGTCAGGGCGCGTCAGCGGG
TGTTGGCGGGTGTCGGGGCGCAGCCATGACCCAGTCACGTAGCGATAGCGGAGTGTATACTGGCTTAACTATGCGGCAT
CAGAGCAGATTGTACTGAGAGTGCACCATATGCGGTGTGAAATACCGCACAGATGCGTAAGGAGAAAATACCGCATCAG
GCGCTCTTCCGCTTCCTCGCTCACTGACTCGCTGCGCTCGGTCGTTCGGCTGCGGCGAGCGGTATCAGCTCACTCAAAG
GCGGTAATACGGTTATCCACAGAATCAGGGGATAACGCAGGAAAGAACATGTGAGCAAAAGGCCAGCAAAAGGCCAGGA
ACCGTAAAAAGGCCGCGTTGCTGGCGTTTTTCCATAGGCTCCGCCCCCCTGACGAGCATCACAAAAATCGACGCTCAAG
TCAGAGGTGGCGAAACCCGACAGGACTATAAAGATACCAGGCGTTTCCCCCTGGAAGCTCCCTCGTGCGCTCTCCTGTT
CCGACCCTGCCGCTTACCGGATACCTGTCCGCCTTTCTCCCTTCGGGAAGCGTGGCGCTTTCTCATAGCTCACGCTGTA
GGTATCTCAGTTCGGTGTAGGTCGTTCGCTCCAAGCTGGGCTGTGTGCACGAACCCCCCGTTCAGCCCGACCGCTGCGC
CTTATCCGGTAACTATCGTCTTGAGTCCAACCCGGTAAGACACGACTTATCGCCACTGGCAGCAGCCACTGGTAACAGG
ATTAGCAGAGCGAGGTATGTAGGCGGTGCTACAGAGTTCTTGAAGTGGTGGCCTAACTACGGCTACACTAGAAGGACAG
TATTTGGTATCTGCGCTCTGCTGAAGCCAGTTACCTTCGGAAAAAGAGTTGGTAGCTCTTGATCCGGCAAACAAACCAC
CGCTGGTAGCGGTGGTTTTTTTGTTTGCAAGCAGCAGATTACGCGCAGAAAAAAAGGATCTCAAGAAGATCCTTTGATC
TTTTCTACGGGGTCTGACGCTCAGTGGAACGAAAACTCACGTTAAGGGATTTTGGTCATGAGATTATCAAAAAGGATCT

TCACCTAGATCCTTTTAAATTAAAAATGAAGTTTTAAATCAATCTAAAGTATATATGAGTAAACTTGGTCTGACAGTTA
CCAATGCTTAATCAGTGAGGCACCTATCTCAGCGATCTGTCTATTTCGTTCATCCATAGTTGCCTGACTCCCCGTCGTG
TAGATAACTACGATACGGGAGGGCTTACCATCTGGCCCCAGTGCTGCAATGATACCGCGAGACCCACGCTCACCGGCTC
CAGATTTATCAGCAATAAACCAGCCAGCCGGAAGGGCCGAGCGCAGAAGTGGTCCTGCAACTTTATCCGCCTCCATCCA
GTCTATTAATTGTTGCCGGGAAGCTAGAGTAAGTAGTTCGCCAGTTAATAGTTTGCGCAACGTTGTTGCCATTGCTGCA
GGCATCGTGGTGTCACGCTCGTCGTTTGGTATGGCTTCATTCAGCTCCGGTTCCCAACGATCAAGGCGAGTTACATGAT
CCCCCATGTTGTGCAAAAAAGCGGTTAGCTCCTTCGGTCCTCCGATCGTTGTCAGAAGTAAGTTGGCCGCAGTGTTATC
ACTCATGGTTATGGCAGCACTGCATAATTCTCTTACTGTCATGCCATCCGTAAGATGCTTTTCTGTGACTGGTGAGTAC
TCAACCAAGTCATTCTGAGAATAGTGTATGCGGCGACCGAGTTGCTCTTGCCCGGCGTCAACACGGGATAATACCGCGC
CACATAGCAGAACTTTAAAAGTGCTCATCATTGGAAAACGTTCTTCGGGGCGAAAACTCTCAAGGATCTTACCGCTGTT
GAGATCCAGTTCGATGTAACCCACTCGTGCACCCAACTGATCTTCAGCATCTTTTACTTTCACCAGCGTTTCTGGGTGA
GCAAAAACAGGAAGGCAAAATGCCGCAAAAAAGGGAATAAGGGCGACACGGAAATGTTGAATACTCATACTCTTCCTTT
TTCAATATTATTGAAGCATTTATCAGGGTTATTGTCTCATGAGCGGATACATATTTGAATGTATTTAGAAAAATAAACA
AATAGGGGTTCCGCGCACATTTCCCCGAAAAGTGCCACCTGACGTCTAAGAAACCATTATTATCATGACATTAACCTAT
AAAAATAGGCGTATCACGAGGCCCTTTCGTCTTCAA

>pMH04

GAATTCTGAACCAGTCCTAAAACGAGTAAATAGGACCGGCAATTCTTCAAGCAATAAACAGGAATACCAATTATTAAAA
GATAACTTAGTCAGATCGTACAATAAAGCTTTGAAGAAAAATGCGCCTTATTCAATCTTTGCTATAAAAAATGGCCCAA
AATCTCACATTGGAAGACATTTGATGACCTCATTTCTTTCAATGAAGGGCCTAACGGAGTTGACTAATGTTGTGGGAAA
TTGGAGCGATAAGCGTGCTTCTGCCGTGGCCAGGACAACGTATACTCATCAGATAACAGCAATACCTGATCACTACTTC
GCACTAGTTTCTCGGTACTATGCATATGATCCAATATCAAAGGAAATGATAGCATTGAAGGATGAGACTAATCCAATTG
AGGAGTGGCAGCATATAGAACAGCTAAAGGGTAGTGCTGAAGGAAGCATACGATACCCCGCATGGAATGGGATAATATC
ACAGGAGGTACTAGACTACCTTTCATCCTACATAAATAGACGCATATAAGTACGCATTTAAGCATAAACACGCACTATG
CCGTTCTTCTCATGTATATATATATACAGGCAACACGCAGATATAGGTGCGACGTGAACAGTGAGCTGTATGTGCGCAG
CTCGCGTTGCATTTTCGGAAGCGCTCGTTTTCGGAAACGCTTTGAAGTTCCTATTCCGAAGTTCCTATTCTCTAGAAAG
TATAGGAACTTCAGAGCGCTTTTGAAAACCAAAAGCGCTCTGAAGACGCACTTTCAAAAAACCAAAAACGCACCGGACT
GTAACGAGCTACTAAAATATTGCGAATACCGCTTCCACAAACATTGCTCAAAAGTATCTCTTTGCTATATATCTCTGTG
CTATATCCCTATATAACCTACCCATCCACCTTTCGCTCCTTGAACTTGCATCTAAACTCGACCTCTACATTTTTTATGT
TTATCTCTAGTATTACTCTTTAGACAAAAAAATTGTAGTAAGAACTATTCATAGAGTGAATCGAAAACAATACGAAAAT
GTAAACATTTCCTATACGTAGTATATAGAGACAAAATAGAAGAAACCGTTCATAATTTTCTGACCAATGAAGAATCATC
AACGCTATCACTTTCTGTTCACAAAGTATGCGCAATCCACATCGGTATAGAATATAATCGGGGATGCCTTTATCTTGAA
AAAATGCACCCGCAGCTTCGCTAGTAATCAGTAAACGCGGGAAGTGGAGTCAGGCTTTTTTTATGGAAGAGAAAATAGA
CACCAAAGTAGCCTTCTTCTAACCTTAACGGACCTACAGTGCAAAAAGTTATCAAGAGACTGCATTATAGAGCGCACAA
AGGAGAAAAAAAGTAATCTAAGATGCTTTGTTAGAAAAATAGCGCTCTCGGGATGCATTTTTGTAGAACAAAAAAGAAG
TATAGATTCTTTGTTGGTAAAATAGCGCTCTCGCGTTGCATTTCTGTTCTGTAAAAATGCAGCTCAGATTCTTTGTTTG
AAAAATTAGCGCTCTCGCGTTGCATTTTTGTTTTACAAAAATGAAGCACAGATTCTTCGTTGGTAAAATAGCGCTTTCG
CGTTGCATTTCTGTTCTGTAAAAATGCAGCTCAGATTCTTTGTTTGAAAAATTAGCGCTCTCGCGTTGCATTTTTGTTC
TACAAAATGAAGCACAGATGCTTCGTTAACAAAGATATGCTATTGAAGTGCAAGATGGAAACGCAGAAAATGAACCGGG
GATGCGACGTGCAAGATTACCTATGCAATAGATGCAATAGTTTCTCCAGGAACCGAAATACATACATTGTCTTCCGTAA
AGCGCTAGACTATATATTATTATACAGGTTCAAATATACTATCTGTTTCAGGGAAAACTCCCAGGTTCGGATGTTCAAA
ATTCAATGATGGGTAACAAGTACGATCGTAAATCTGTAAAACAGTTTGTCGGATATTAGGCTGTATCTCCTCAAAGCGT
ATTCGAATATCATTGAGAAGCTGCAGCGTCACATCGGATAATAATGATGGCAGCCATTGTAGAAGTGCCTTTTGCATTT
CTAGTCTCTTTCTCGGTCTAGCTAGTTTTACTACATCGCGAAGATAGAATCTTAGATCACACTGCCTTTGCTGAGCTGG
ATCAATAGAGTAACAAAAGAGTGGTAAGGCCTCGTTAAAGGACAAGGACCTGAGCGGAAGTGTATCGTACAGTAGACGG
AGTATACTAGTATAGTCTATAGTCCGTGGAATTCTCATGTTTGACAGCTTATCATCGATAAGCTTTTCAATTCAATTCA
TCATTTTTTTTTTTATTCTTTTTTTTTGATTTCGGTTTCTTTGAAATTTTTTTGATTCGGTAATCTCCGAACAGAAGGAAG
AACGAAGGAAGGAGCACAGACTTAGATTGGTATATATACGCATATGTAGTGTTGAAGAAACATGAAATTGCCCAGTATT
CTTAACCCAACTGCACAGAACAAAAACCTGCAGGAAACGAAGATAAATCATGTCGAAAGCTACATATAAGGAACGTGCT
GCTACTCATCCTAGTCCTGTTGCTGCCAAGCTATTTAATATCATGCACGAAAAGCAAACAAACTTGTGTGCTTCATTGG
ATGTTCGTACCACCAAGGAATTACTGGAGTTAGTTGAAGCATTAGGTCCCAAAATTTGTTTACTAAAAACACATGTGGA
TATCTTGACTGATTTTTCCATGGAGGGCACAGTTAAGCCGCTAAAGGCATTATCCGCCAAGTACAATTTTTTACTCTTC
GAAGACAGAAAATTTGCTGACATTGGTAATACAGTCAAATTGCAGTACTCTGCGGGTGTATACAGAATAGCAGAATGGG

CAGACATTACGAATGCACACGGTGTGGTGGGCCCAGGTATTGTTAGCGGTTTGAAGCAGGCGGCAGAAGAAGTAACAAA
GGAACCTAGAGGCCTTTTGATGTTAGCAGAATTGTCATGCAAGGGCTCCCTATCTACTGGAGAATATACTAAGGGTACT
GTTGACATTGCGAAGAGCGACAAAGATTTTGTTATCGGCTTTATTGCTCAAAGAGACATGGGTGGAAGAGATGAAGGTT
ACGATTGGTTGATTATGACACCCGGTGTGGGTTTAGATGACAAGGGAGACGCATTGGGTCAACAGTATAGAACCGTGGA
TGATGTGGTCTCTACAGGATCTGACATTATTATTGTTGGAAGAGGACTATTTGCAAAGGGAAGGGATGCTAAGGTAGAG
GGTGAACGTTACAGAAAAGCAGGCTGGGAAGCATATTTGAGAAGATGCGGCCAGCAAAACTAAAAAACTGTATTATAAG
TAAATGCATGTATACTAAACTCACAAATTAGAGCTTCAATTTAATTATATCAGTTATTACCCGGGAATCTCGGTCGTAA
TGATTTTTATAATGACGAAAAAAAAAAAAATTGGAAAGAAAAAGCTTTAATGCGGTAGTTTATCACAGTTAAATTGCTAA
CGCAGTCAGGCACCGTGTATGAAATCTAACAATGCGCTCATCGTCATCCTCGGCACCGTCACCCTGGATGCTGTAGGCA
TAGGCTTGGTTATGCCGGTACTGCCGGGCCTCTTGCGGGATATCGTCCATTCCGACAGCATCGCCAGTCACTATGGCGT
GCTGCTAGCGCTATATGCGTTGATGCAATTTCTATGCGCACCCGTTCTCGGAGCACTGTCCGACCGCTTTGGCCGCCGC
CCAGTCCTGCTCGCTTCGCTACTTGGAGCCACTATCGACTACGCGATCATGGCGACCACACCCGTCCTGTGGATCCTCT
ACGCCGGACGCATCGTGGCCGGCATCACCGGCGCCACAGGTGCGGTTGCTGGCGCCTATATCGCCGACATCACCGATGG
GGAAGATCGGGCTCGCCACTTCGGGCTCATGAGCGCTTGTTTCGGCGTGGGTATGGTGGCAGGCCCCGTGGCCGGGGGA
CTGTTGGGCGCCATCTCCTTGCATGCACCATTCCTTGCGGCGGCGGTGCTCAACGGCCTCAACCTACTACTGGGCTGCT
TCCTAATGCAGGAGTCGCATAGCCAGTGTGATGGATATCTGCAGAATTCGCCCTTacctacccatgtctagaaagagcc
aatcagaatcaagtgaggctaccattacttcaaatagatttatttccgaagatccaagtaaaccggtgagagaaatttc
cgatattgaattatcgcaatttttagaagagtcggtacattttaaaaaaaagtatttaggtggtgttcattttgtatt
aataatattcgttaaattttgtgtatactttacttaagctttttcttttaagattttagtaacagcttcgacaatcgcg
tcaccaacttcagtagctgaactggtacctcttaaatctgcagttctgatacctttatccaagacttgtttcacggctt
cttctaaagcttcagcttctttcacacaatccaaagataatctcaacatagaagcagcagataaaatagtagcaattgg
gttaactttattggctggtaaatcaggagcagaaccgtgacatggttcataaagaccaaaagcggtgttggtatctggc
aatgaagccaaagatgcagatggtaataaacccaaggaacctggaataactgaagcttcatcggaaatgatatcaccaa
acatgttagaagtgatgattataccattcaatttggttgggttttgaattaaaatcatggcggcggagtcaatcaattg
atgttgaacagacaaagttgggaactcttcagaaatcactttatcgacagttcttctccataatctagaagaagctaaa
acattagccttatccaatgaccagattggtaatggtgggttatgttgtaaagccatgaaggcagccatacgggtgattc
tggtgacttcatcgacggtgtatttttcagtatcccatgcggtttgtttatcttcactttcttcttgttcttcacgatc
accgaaataaataccaccaactaattcacgaacaatgatcaagtttgtacctttaacgacttctggtctcaatggggat
aattccaataaggagtcactggcaaagttacatggtctgatgttagcataaaggttcaattctttacgaatctttaata
aaccttgttctggacgaacggcgccagtcccccattttggtccaccaacagcacccaataagacggcatcagatttttt
ggcactttcaagtgcgtcatctggcaatgggacaccagtggcatcaatggcagcaccaccgattaaatgatgtttgaaa
tcgaaatggatttttttggtaaggagtggcagcttcaattgcttctaaaactttgatagcttcattgacaatttcggtac
cgacgtgatcacctggtaaaattgtaatagttttagttttaactgacattgtgggtttgggtagtgaataaaggaatta
aggatgaaggaaaaaaaattcttgagtgaaaaatttcatagcctgagtccctcggatttttttcgtttattatatattt
ttatagtgaacaagaaaaaaaaaaattgaacccggctgcggaacccacgcacgccgttacaggagttagtcattcttcaa
caatacgaacgtattattcatctggttcagatacatgaacgaaagtacaatttccgaaattactctatacacgataatt
acacataacaacacaaattacaaaaaaacaaaaaaccttaatgtccactccattcgatagagttcaagtcaataccttc
ttgaaccatttcccataatggagacatctttctcaagaaatcaactctttcattaattgcttgaataacatagtcaact

tctgcttcagtggtgaatctaccaataccgaatctaatggaagaatgagccaaggcatcatcggcacccaaagcatgta
aaacatacgatggttccaaagatgcagaggtacatgcagaaccagaactcaatgcaatatctttcaatgccattaacaa
agattccccttcaatgtatgcaaaggaaacattcacacaacctgggtattgatatgttgggttattacaaccgttaaac
tgagtagatggaatggataacaaaccgttcttcaatttagtagataacctctcaatatgtttcttgtcaaatgcagatt
cttgtttcattaatcttgcagcttcaccaaacccagctactaatggtggtgccaaagtacctgatctcaaacctctttc
ttgacccccaccagtgatgattggatccAAGGGCGAATTCCAGCACACTGGCGGTGAGAGCCTTCAACCCAGTCAGCTC
CTTCCGGTGGGCGCGGGGCATGACTATCGTCGCCGCACTTATGACTGTCTTCTTTATCATGCAACTCGTAGGACAGGTG
CCGGCAGCGCTCTGGGTCATTTTCGGCGAGGACCGCTTTCGCTGGAGCGCGACGATGATCGGCCTGTCGCTTGCGGTAT
TCGGAATCTTGCACGCCCTCGCTCAAGCCTTCGTCACTGGTCCCGCCACCAAACGTTTCGGCGAGAAGCAGGCCATTAT
CGCCGGCATGGCGGCCGACGCGCTGGGCTACGTCTTGCTGGCGTTCGCGACGCGAGGCTGGATGGCCTTCCCCATTATG
ATTCTTCTCGCTTCCGGCGGCATCGGGATGCCCGCGTTGCAGGCCATGCTGTCCAGGCAGGTAGATGACGACCATCAGG
GACAGCTTCAAGGATCGCTCGCGGCTCTTACCAGCCTAACTTCGATCACTGGACCGCTGATCGTCACGGCGATTTATGC
CGCCTCGGCGAGCACATGGAACGGGTTGGCATGGATTGTAGGCGCCGCCCTATACCTTGTCTGCCTCCCCGCGTTGCGT
CGCGGTGCATGGAGCCGGGCCACCTCGACCTGAATGGAAGCCGGCGGCACCTCGCTAACGGATTCACCACTCCAAGAAT
TGGAGCCAATCAATTCTTGCGGAGAACTGTGAATGCGCAAACCAACCCTTGGCAGAACATATCCATCGCGTCCGCCATC
TCCAGCAGCCGCACGCGGCGCATCTCGGGCAGCGTTGGGTCCTGGCCACGGGTGCGCATGATCGTGCTCCTGTCGTTGA
GGACCCGGCTAGGCTGGCGGGGTTGCCTTACTGGTTAGCAGAATGAATCACCGATACGCGAGCGAACGTGAAGCGACTG
CTGCTGCAAAACGTCTGCGACCTGAGCAACAACATGAATGGTCTTCGGTTTCCGTGTTTCGTAAAGTCTGGAAACGCGG
AAGTCAGCGCCCTGCACCATTATGTTCCGGATCTGCATCGCAGGATGCTGCTGGCTACCCTGTGGAACACCTACATCTG
TATTAACGAAGCGCTGGCATTGACCCTGAGTGATTTTTCTCTGGTCCCGCCGCATCCATACCGCCAGTTGTTTACCCTC
ACAACGTTCCAGTAACCGGGCATGTTCATCATCAGTAACCCGTATCGTGAGCATCCTCTCTCGTTTCATCGGTATCATT
ACCCCCATGAACAGAAATTCCCCCTTACACGGAGGCATCAAGTGACCAAACAGGAAAAAACCGCCCTTAACATGGCCCG
CTTTATCAGAAGCCAGACATTAACGCTTCTGGAGAAACTCAACGAGCTGGACGCGGATGAACAGGCAGACATCTGTGAA
TCGCTTCACGACCACGCTGATGAGCTTTACCGCAGCTGCCTCGCGCGTTTCGGTGATGACGGTGAAAACCTCTGACACA
TGCAGCTCCCGGAGACGGTCACAGCTTGTCTGTAAGCGGATGCCGGGAGCAGACAAGCCCGTCAGGGCGCGTCAGCGGG
TGTTGGCGGGTGTCGGGGCGCAGCCATGACCCAGTCACGTAGCGATAGCGGAGTGTATACTGGCTTAACTATGCGGCAT
CAGAGCAGATTGTACTGAGAGTGCACCATATGCGGTGTGAAATACCGCACAGATGCGTAAGGAGAAAATACCGCATCAG
GCGCTCTTCCGCTTCCTCGCTCACTGACTCGCTGCGCTCGGTCGTTCGGCTGCGGCGAGCGGTATCAGCTCACTCAAAG
GCGGTAATACGGTTATCCACAGAATCAGGGGATAACGCAGGAAAGAACATGTGAGCAAAAGGCCAGCAAAAGGCCAGGA
ACCGTAAAAAGGCCGCGTTGCTGGCGTTTTTCCATAGGCTCCGCCCCCCTGACGAGCATCACAAAAATCGACGCTCAAG
TCAGAGGTGGCGAAACCCGACAGGACTATAAAGATACCAGGCGTTTCCCCCTGGAAGCTCCCTCGTGCGCTCTCCTGTT
CCGACCCTGCCGCTTACCGGATACCTGTCCGCCTTTCTCCCTTCGGGAAGCGTGGCGCTTTCTCATAGCTCACGCTGTA
GGTATCTCAGTTCGGTGTAGGTCGTTCGCTCCAAGCTGGGCTGTGTGCACGAACCCCCCGTTCAGCCCGACCGCTGCGC
CTTATCCGGTAACTATCGTCTTGAGTCCAACCCGGTAAGACACGACTTATCGCCACTGGCAGCAGCCACTGGTAACAGG
ATTAGCAGAGCGAGGTATGTAGGCGGTGCTACAGAGTTCTTGAAGTGGTGGCCTAACTACGGCTACACTAGAAGGACAG
TATTTGGTATCTGCGCTCTGCTGAAGCCAGTTACCTTCGGAAAAAGAGTTGGTAGCTCTTGATCCGGCAAACAAACCAC
CGCTGGTAGCGGTGGTTTTTTTGTTTGCAAGCAGCAGATTACGCGCAGAAAAAAAGGATCTCAAGAAGATCCTTTGATC
TTTTCTACGGGGTCTGACGCTCAGTGGAACGAAAACTCACGTTAAGGGATTTTGGTCATGAGATTATCAAAAAGGATCT

TCACCTAGATCCTTTTAAATTAAAAATGAAGTTTTAAATCAATCTAAAGTATATATGAGTAAACTTGGTCTGACAGTTA
CCAATGCTTAATCAGTGAGGCACCTATCTCAGCGATCTGTCTATTTCGTTCATCCATAGTTGCCTGACTCCCCGTCGTG
TAGATAACTACGATACGGGAGGGCTTACCATCTGGCCCCAGTGCTGCAATGATACCGCGAGACCCACGCTCACCGGCTC
CAGATTTATCAGCAATAAACCAGCCAGCCGGAAGGGCCGAGCGCAGAAGTGGTCCTGCAACTTTATCCGCCTCCATCCA
GTCTATTAATTGTTGCCGGGAAGCTAGAGTAAGTAGTTCGCCAGTTAATAGTTTGCGCAACGTTGTTGCCATTGCTGCA
GGCATCGTGGTGTCACGCTCGTCGTTTGGTATGGCTTCATTCAGCTCCGGTTCCCAACGATCAAGGCGAGTTACATGAT
CCCCCATGTTGTGCAAAAAAGCGGTTAGCTCCTTCGGTCCTCCGATCGTTGTCAGAAGTAAGTTGGCCGCAGTGTTATC
ACTCATGGTTATGGCAGCACTGCATAATTCTCTTACTGTCATGCCATCCGTAAGATGCTTTTCTGTGACTGGTGAGTAC
TCAACCAAGTCATTCTGAGAATAGTGTATGCGGCGACCGAGTTGCTCTTGCCCGGCGTCAACACGGGATAATACCGCGC
CACATAGCAGAACTTTAAAAGTGCTCATCATTGGAAAACGTTCTTCGGGGCGAAAACTCTCAAGGATCTTACCGCTGTT
GAGATCCAGTTCGATGTAACCCACTCGTGCACCCAACTGATCTTCAGCATCTTTTACTTTCACCAGCGTTTCTGGGTGA
GCAAAAACAGGAAGGCAAAATGCCGCAAAAAAGGGAATAAGGGCGACACGGAAATGTTGAATACTCATACTCTTCCTTT
TTCAATATTATTGAAGCATTTATCAGGGTTATTGTCTCATGAGCGGATACATATTTGAATGTATTTAGAAAAATAAACA
AATAGGGGTTCCGCGCACATTTCCCCGAAAAGTGCCACCTGACGTCTAAGAAACCATTATTATCATGACATTAACCTAT
AAAAATAGGCGTATCACGAGGCCCTTTCGTCTTCAA
